# Investigation of the trends and associated factors of ovarian cancer in Indonesia: A systematic analysis of the Global Burden of Disease study 1990–2021

**DOI:** 10.1371/journal.pone.0313418

**Published:** 2025-01-17

**Authors:** Brahmana Askandar Tjokroprawiro, Khoirunnisa Novitasari, Renata Alya Ulhaq, Hanif Ardiansyah Sulistya, Santi Martini

**Affiliations:** 1 Department of Obstetrics and Gynecology, Faculty of Medicine, Universitas Airlangga/Dr. Soetomo General Academic Hospital, Surabaya, Indonesia; 2 ARC Institute, Surabaya, Indonesia; 3 Departement of Epidemiology, Faculty of Public Health, Universitas Airlangga, Surabaya, Indonesia; Kerman University of Medical Sciences, ISLAMIC REPUBLIC OF IRAN

## Abstract

**Introduction:**

Ovarian cancer is one of the most lethal gynecological cancers. Despite diagnosis and treatment advances, survival rates have not increased over the past 32 years. This study estimated and reported the global burden of ovarian cancer during the past 32 years to inform preventative and control strategies.

**Methods:**

We examined ovarian cancer incidence, mortality, and disability-adjusted life years (DALYs) using age-standardized rates from the Global Burden of Disease, Injuries, and Risk Factors Study 2021. high body mass index and occupational asbestos exposure were linked with death and DALYs. Data are presented as averages with 95% uncertainty intervals (UIs).

**Results:**

Indonesia had 13 250 (8 574–21 565) ovarian cancer cases in 2021, with 5 296 (3 520–8958) deaths and 186 917 (121 866–309 820) DALYs. The burden increased by 233.53% for new cases, 221.95% for mortalities, and 206.65% for DALYs. The age-standardized rate also increased from 1990 to 2021. Ovarian cancer burden increased with age but declined in the 50+ year age group. According to the sociodemographic index, the gross domestic product per capita and number of obstetricians and oncologic gynecologists in provinces showed different trends.

**Conclusions:**

Indonesian ovarian cancer rates are rising despite gynecologic oncologists in 24 of 34 provinces. These findings will help policymakers and healthcare providers identify ovarian cancer prevention and control gaps.

## Introduction

Ovarian cancer (OC), a highly lethal type of gynecologic cancer, poses a substantial global health threat [1]. As per the most recent worldwide cancer burden statistics from 2020, it is the eighth most prevalent cause of cancer-related fatalities in women. The situation is particularly severe in regions such as Australia, North America, and Western Europe, where OC is the fifth leading cause of cancer-related deaths among women. Globally, it is responsible for approximately 5% of all deaths due to female cancers, a proportion that is higher than that for any other gynecologic cancer [2]. According to GLOBOCAN 2022, ovarium cancer is the third most common female cancer in Indonesia [3]. Challenges exist in the initial identification, diagnostic procedures, therapeutic interventions, and overall survival outcomes for individuals with malignant OC [4]. For instance, in Indonesia, OC’s 5-year cumulative survival rate in Indonesia is 54.8% [5]. The persistent prevalence of OC underscores the urgent need for continued investigations into its prevention, early diagnosis, and development of more effective therapeutic approaches.

From a global standpoint today, the prevalence of OC differs substantially across nations. This variability and the complex nature of OC underscores its emergence as a worldwide public health issue. It is essential to identify pertinent global patterns via statistical evaluation and analysis to understand the extensive impact of this disease on public health [6].

Several risk factors contribute to OC, including smoking history, alcohol consumption, and age at menarche [7, 8]. However, comprehensive reviews encompassing global data on OC risk factors are scarce. Even when such reviews exist, they often include studies that report no such associations. Although previous studies have examined the Global Burden of Diseases (GBD) 2020 data for OC, an updated review is crucial [6, 9]. Any considerable shifts in the disease burden require scrutiny. A focused analysis at Indonesia’s provincial level is essential to address aggregation biases in previous national-level data. As shown in some studies, the trends in absolute figures might contradict the trends in related age-standardized rates over similar periods, as observed in cardiovascular diseases and unintentional injuries, e.g., burn injuries [10, 11]. Consequently, it is imperative to consider absolute numbers alongside age-standardized rates.

This study aimed to fill a substantial gap in the literature by providing a comprehensive analysis of the distribution and temporal trends in OC burden at both the national and provincial levels in Indonesia, covering the period from 1990 to 2021, using a dataset previously unavailable, in contrast to prior GB investigations on OC, which primarily examined geographical and longitudinal patterns in incidence and mortality [6, 9]. The objectives of the current research extend beyond these aspects. Specifically, we aimed to estimate the number of newly diagnosed OC cases, associated mortalities, and corresponding disability-adjusted life years (DALYs) between 1990 and 2021, categorized by province, age group, and sociodemographic status. Additionally, we intended to scrutinize age-specific trends in OC and identify the principal risk factors and availability of healthcare personnel contributing to OC fatalities.

## Methods

### Study design and population

This study derived data from the Global Burden of Diseases, Injuries, and Risk Factors Study 2021 (GBD 2021), a comprehensive tool used to quantify health detriments from many diseases, injuries, and risk factors to enhance health systems and eradicate disparities [12]. The GBD study has gained wide recognition for its utility in comprehending the global disease burden, especially in oncology [13]. The search for data on OC at the national and provincial levels in women from 1990 to 2021 was conducted through the GBD study as derived from the ICD-9 (B123) and ICD-10 (C56) of the International Classification of Diseases, which adheres to the Guidelines for Accurate and Transparent Health Estimates Reporting (GATHER) [14]. The Institute for Health Metrics and Evaluation maintains the results tool, a comprehensive health-related data repository available on Global Health Data Exchange (http://ghdx.healthdata.org).

Incidence, mortality, and disability-adjusted life years, along with their age-standardized rates, were chosen as parameters for evaluating disease burden. The data were categorized by geographic location, age groups, and associated risk factors. The specific study design and methods of the GBD study are extensively documented in prior publications [12, 13] In this section, we provided a brief explanation of the methodologies applied to evaluate the burden of OC and its associated risk factors.

Furthermore, countries and regions were classified based on geographical attributes and their sociodemographic index (SDI) and stratified into four distinct categories: high, middle, low-middle, and low SDI. Measured on a continuum ranging from 0 to 1, the SDI functions as an integrative metric that captures the socio-economic milieu of a specified geographical entity. This index incorporates per capita income, educational levels for individuals older than 15, and fertility rates for those aged 25. Empirical evidence has suggested that the SDI correlates robustly with various health outcomes [15–18].

### Statistical analysis

The GBD study enhances data availability by separately modeling mortality-to-incidence ratios (MIRs) using cancer registry data. These mortality figures then feed into the Cause of Death Ensemble Model (CODEm), which uses existing data and causal covariates to forecast single-cause mortality, providing estimates of OC mortality by demographic and temporal variables [12]. This modeling approach has been detailed in previous studies [19].

For incidence and disability analysis in the GBD 2021 study, OC incidence is derived from a comprehensive review of microdata from cohorts and registries, as well as macro-administrative data, analyzed using Bayesian meta-regression software DisMod-MR 2.1 [20]. Disability-adjusted life years (DALYs) are calculated by summing the years of life lost (YLL) due to premature death and years lived with disability (YLD) from nonfatal health deficits, with estimation methods for both YLL and YLD described in prior research [21]. This approach addresses the variability and inconsistencies often found in epidemiological data, ensuring a robust measure of disease impact. 95% Uncertainty Interval (UI) for each metric are determined by the 25th and 975th ranked values from 1000 samples of the posterior distribution.

We conducted a descriptive analysis to assess the impact of OC in Indonesia. This involved comparing the estimates of incidence, mortality, and DALYs. The study further delved into the burden of OC across Indonesian provinces, categorizing the data based on the highest and lowest age-standardized DALY rates per 100,000 individuals and the incidence and prevalence rates. The correlation between OC metrics and the SDI, number of obstetric gynecologists, and number of gynecologic oncologists was analyzed using R version 4.1.2 (R Core Team). Results were considered statistically significant at a p-value of <0.05. Then, the findings were illustrated using a scatter plots and visualized using Tableau version 2022.4 Software (Salesforce Inc.). Meanwhile, the choropleth maps were created using Datawrapper (https://www.datawrapper.de/) with license CC BY 4.0 [22].

### Ethics statement

This research did not require permission from an Ethics committee because it utilized solely data from publicly accessible secondary databases.

### Patient and public involvement statement

It was neither suitable nor feasible to include patients or the general public in the research’s, conception, implementation, reporting, or dissemination plans.

## Results

The number of OC incidence cases nationwide increased from 3973 (95% uncertainty interval [UI], 2716–7573) to 13 250 (95% UI, 8574–21 565) for the past 32 years. The overall burden of OC was at an all-time high, especially in the number of cases, with a percentage change of 233.53% compared to 1990 (**Table 1)**. The age-specific incidence rate (ASIR) also demonstrated an upward trend (EAPC: 1.22). In 2021, the prevalence of OC was 63 955 cases. Most cases were distributed on Java Island. In all SDI quintiles, the prevalence of OC in the middle-high SDI quintile provinces (27 921) ranked first, followed by the low SDI quintile provinces (15 568).

**Table 1:** Ovarian cancer incidence, mortality, and DALYs by provincial level in Indonesia, 1990–2021.

Province	Number of Incidence				Number of Mortality				Number of DALYs			
	1990	2021	Change (%)	AAPC (%)	1990	2021	Change (%)	AAPC (%)	1990	2021	Change (%)	AAPC (%)
Indonesia	3972.85 (2716.45–7572.62)	13250.48 (8573.93–21564.96)	233.53	3.72	1645.03 (1176.19–3051.40)	5296.21 (3519.94–8958.22)	221.95	3.65	60954.96 (42515.96–114826.65)	186917.18 (121865.63–309820.43)	206.65	3.51
Aceh	59.0 (35.47–126.26)	245.71 (137.81–460.65)	316.46	4.44	24.08 (14.62–50.50)	97.14 (53.89–183.11)	303.33	4.41	903.80 (539.25–1888.03)	3492.53 (1954.70–6513.31)	286.43	4.28
Bali	85.67 (53.53–191.13)	258.08 (153.17–444.41)	201.25	3.44	35.28 (22.71–77.47)	106.33 (62.75–188.20)	201.37	3.51	1274.21 (819.54–2818.23)	3552.47 (2084.89–6299.73)	178.80	3.28
Bangka-Belitung Islands	16.58 (8.95–46.43)	70.85 (37.85–168.19)	327.32	4.52	6.70 (3.73–18.07)	27.28 (15.17–60.70)	307.16	4.43	254.59 (139.96–702.0)	1006.37 (542.02–2329.94)	295.29	4.36
Banten	148.20 (89.84–287.14)	552.38 (333.70–908.22)	272.73	3.67	56.37 (36.0–103.77)	209.05 (127.80–358.37)	270.88	3.69	2247.92 (1393.22–4251.62)	7870.38 (4750.91–13172.54)	250.12	3.55
Bengkulu	16.39 (8.72–45.50)	88.67 (48.49–184.10)	441.00	5.32	6.72 (3.83–17.88)	35.15 (19.71–75.30)	423.08	5.25	255.34 (139.63–695.66)	1278.40 (703.22–2731.60)	400.67	5.14
Central Java	684.72 (458.58–1130.68)	1848.52 (1115.84–3097.92)	169.97	3.06	291.19 (203.33–464.67)	766.79 (476.09–1276.34)	163.33	3.02	10483.12 (7242.46–17098.71)	25995.82 (15785.10–44730.08)	147.98	2.85
Central Kalimantan	24.21 (13.48–57.80)	134.47 (77.23–248.13)	455.43	5.25	9.31 (5.40–21.71)	51.12 (30.85–94.83)	449.36	5.36	365.31 (207.85–876.64)	1891.61 (1120.44–3499.93)	417.81	5.14
Central Sulawesi	27.69 (15.92–75.32)	135.64 (71.90–312.17)	389.85	5.00	10.98 (6.46–27.81)	53.53 (29.57–119.36)	387.61	5.09	433.42 (254.76–1126.16)	1972.25 (1037.11–4461.01)	355.04	4.84
East Java	915.66 (608.09–1763.41)	2220.87 (1274.77–3736.22)	142.54	2.72	405.21 (276.55–776.72)	943.90 (553.03–1580.12)	132.94	2.59	14490.52 (9671.10–28086.84)	31658.65 (17979.69–52841.19)	118.48	2.41
East Kalimantan	32.96 (17.61–96.71)	190.99 (93.38–476.31)	479.46	5.65	11.37 (6.56–31.66)	69.17 (35.53–163.44)	508.57	5.94	471.18 (257.21–1332.25)	2630.83 (1293.89–6350.26)	458.35	5.63
East Nusa Tenggara	70.45 (41.03–147.52)	241.64 (141.05–398.62)	243.00	3.78	31.77 (18.62–66.03)	98.74 (60.13–155.17)	210.82	3.55	1139.51 (666.35–2384.03)	3457.54 (2049.41–5536.24)	203.42	3.46
Gorontalo	14.22 (7.92–40.10)	64.14 (33.28–160.40)	351.05	5.01	5.87 (3.39–15.17)	25.24 (13.49–59.55)	330.02	4.81	221.48 (127.04–585.93)	927.14 (487.87–2229.78)	318.61	4.80
Jakarta	199.59 (118.05–412.72)	547.02 (314.68–1015.22)	174.07	3.12	65.43 (41.50–129.14)	207.65 (117.0–406.55)	217.36	3.70	2742.09 (1687.75–5445.25)	7500.46 (4196.24–14194.49)	173.53	3.20
Jambi	35.77 (20.11–90.98)	163.0 (92.73–329.40)	355.69	4.71	13.31 (8.04–31.51)	63.22 (36.20–123.29)	375.13	4.95	532.36 (299.55–1297.14)	2307.56 (1315.08–4566.77)	333.46	4.64
Lampung	85.10 (52.71–131.60)	396.52 (235.90–616.24)	365.95	4.76	33.36 (21.47–50.32)	157.38 (96.04–241.64)	371.80	4.85	1291.24 (825.28–1957.62)	5607.77 (3369.86–8629.39)	334.29	4.60
Maluku	25.88 (13.09–68.63)	91.01 (50.12–194.13)	251.66	3.60	10.85 (6.06–26.95)	35.20 (20.56–74.86)	224.47	3.45	405.08 (210.97–1053.44)	1301.99 (729.82–2784.27)	221.42	3.43
North Kalimantan	4.33 (2.60–6.63)	25.92 (11.84–43.68)	498.61	5.94	1.42 (.88–2.10)	8.72 (4.18–14.61)	513.88	6.10	57.94 (35.52–88.03)	325.64 (151.29–543.64)	462.03	5.82
North Maluku	12.16 (5.95–35.49)	61.40 (32.03–163.48)	404.93	5.05	5.01 (2.70–13.92)	23.40 (12.32–61.89)	366.96	4.87	193.18 (98.52–555.68)	897.72 (464.54–2357.11)	364.71	4.86
North Sulawesi	57.88 (34.50–137.03)	150.36 (87.37–291.11)	159.78	2.75	24.20 (15.39–55.21)	64.81 (39.21–126.14)	167.77	2.96	888.26 (538.35–2108.61)	2194.65 (1303.97–4160.97)	147.07	2.69
North Sumatra	185.97 (117.67–377.81)	691.50 (413.19–1238.14)	271.83	4.04	76.03 (49.47–142.93)	270.86 (165.89–477.76)	256.23	3.95	2834.30 (1828.21–5490.98)	9740.20 (5873.95–17243.62)	243.65	3.86
Papua	18.50 (7.96–51.74)	219.42 (120.38–555.19)	1086.05	8.17	6.36 (3.18–17.32)	81.39 (45.30–192.18)	1180.06	8.47	275.51 (128.09–757.41)	3243.67 (1794.44–7814.65)	1077.33	8.25
Riau	38.08 (22.61–87.15)	270.02 (153.18–479.77)	609.09	6.47	14.51 (9.06–32.46)	97.36 (56.59–183.01)	570.77	6.34	571.71 (346.50–1313.18)	3702.71 (2128.63–6786.88)	547.66	6.24
Riau Islands	16.01 (9.20–39.77)	91.43 (51.26–187.62)	471.08	5.32	6.01 (3.59–14.96)	33.45 (19.23–71.81)	456.40	5.20	238.05 (139.86–595.74)	1272.90 (714.93–2687.22)	434.72	5.14
South Kalimantan	52.04 (30.50–129.52)	187.43 (109.04–398.32)	260.17	3.96	21.52 (12.88–50.23)	77.26 (45.93–159.38)	259.08	4.04	820.86 (488.78–1979.49)	2770.34 (1618.31–5729.99)	237.49	3.83
South Sulawesi	142.61 (86.92–307.44)	467.61 (287.64–810.82)	227.89	3.67	60.06 (38.22–120.76)	189.44 (117.84–334.61)	215.43	3.57	2218.71 (1382.55–4604.63)	6542.38 (4003.08–11382.80)	194.87	3.37
South Sumatra	88.76 (56.81–151.65)	372.55 (218.84–609.71)	319.73	4.56	34.06 (22.71–57.17)	144.37 (88.69–235.77)	323.93	4.65	1315.01 (861.35–2237.23)	5206.63 (3105.86–8302.52)	295.94	4.44
Southeast Sulawesi	23.99 (12.92–68.63)	116.87 (63.02–256.38)	387.16	4.95	9.62 (5.56–26.06)	45.29 (25.43–94.48)	370.82	4.92	376.72 (209.70–1058.75)	1680.03 (918.54–3562.25)	345.96	4.72
West Java	569.22 (379.12–1069.96)	2240.21 (1381.27–3639.63)	293.56	4.29	230.62 (156.15–422.15)	866.25 (550.32–1378.0)	275.61	4.20	8629.84 (5789.85–15988.24)	31136.58 (19692.88–50453.92)	260.80	4.08
West Kalimantan	52.39 (28.79–129.43)	231.22 (131.97–445.12)	341.34	4.60	20.31 (11.64–48.64)	88.18 (52.90–170.70)	334.25	4.63	803.38 (448.09–1962.58)	3245.65 (1877.0–6316.13)	304.00	4.38
West Nusa Tenggara	61.34 (33.28–159.94)	253.83 (145.33–508.99)	313.81	4.45	25.81 (14.62–64.16)	100.73 (58.56–199.93)	290.25	4.30	974.10 (537.39–2503.56)	3674.71 (2112.60–7340.71)	277.24	4.18
West Papua	10.75 (4.29–31.02)	60.61 (28.76–162.55)	463.81	5.31	3.50 (1.63–10.24)	20.98 (10.64–56.87)	499.06	5.65	155.82 (67.38–461.75)	842.73 (417.12–2272.06)	440.84	5.25
West Sulawesi	14.52 (7.55–44.90)	67.59 (35.79–151.22)	365.50	4.91	5.91 (3.20–17.35)	25.38 (13.97–55.26)	329.16	4.71	231.85 (119.09–708.69)	959.07 (510.65–2100.94)	313.66	4.57
West Sumatra	87.05 (55.93–175.34)	273.63 (162.88–443.87)	214.34	3.57	39.66 (26.08–74.40)	113.43 (69.40–193.46)	185.99	3.28	1389.96 (903.19–2701.99)	3880.09 (2281.55–6383.54)	179.15	3.23
Yogyakarta	95.15 (58.75–211.59)	219.35 (128.53–402.96)	130.53	2.58	42.64 (26.99–93.47)	98.01 (59.70–185.18)	129.88	2.60	1468.55 (916.12–3268.10)	3149.70 (1857.49–5852.99)	114.48	2.41

In the table, dark red signifies metrics substantially above, and dark blue significantly below, the national average; lighter shades of red and blue indicate slight deviations. Grey indicates metrics at or near the national average.

OC cases increased in 34 Indonesian provinces (**S1 Fig**). The incidence in Papua increased most from 18.5 to 219.42. Compared with other provinces in 2021, West Java (2240, 95% UI: 1381–3640) had the highest number of incidences, whereas North Kalimantan (26, 95% UI: 12–44) had the fewest cases. In contrast, West Papua had the highest ASIR (11.2 per 100 000 people), whereas North Kalimantan had the lowest ASIR (7.79 per 100 000 people) in 2021. All age groups showed an increasing trend in the number of cases between 1990 and 2021, with the highest number of cases in 2021 found in the 50–54- year-old age group (1759, 95% UI, 1088–2937) and the lowest found in the +95-year-old age group (3, 95% UI, 2–5) (**S2 Fig)**. The highest change from 1990 to 2021 was observed in the +95-year-old age group (417%), whereas the lowest change was observed in the 15–19- year-old age group (92.49%).

**Fig 1:**
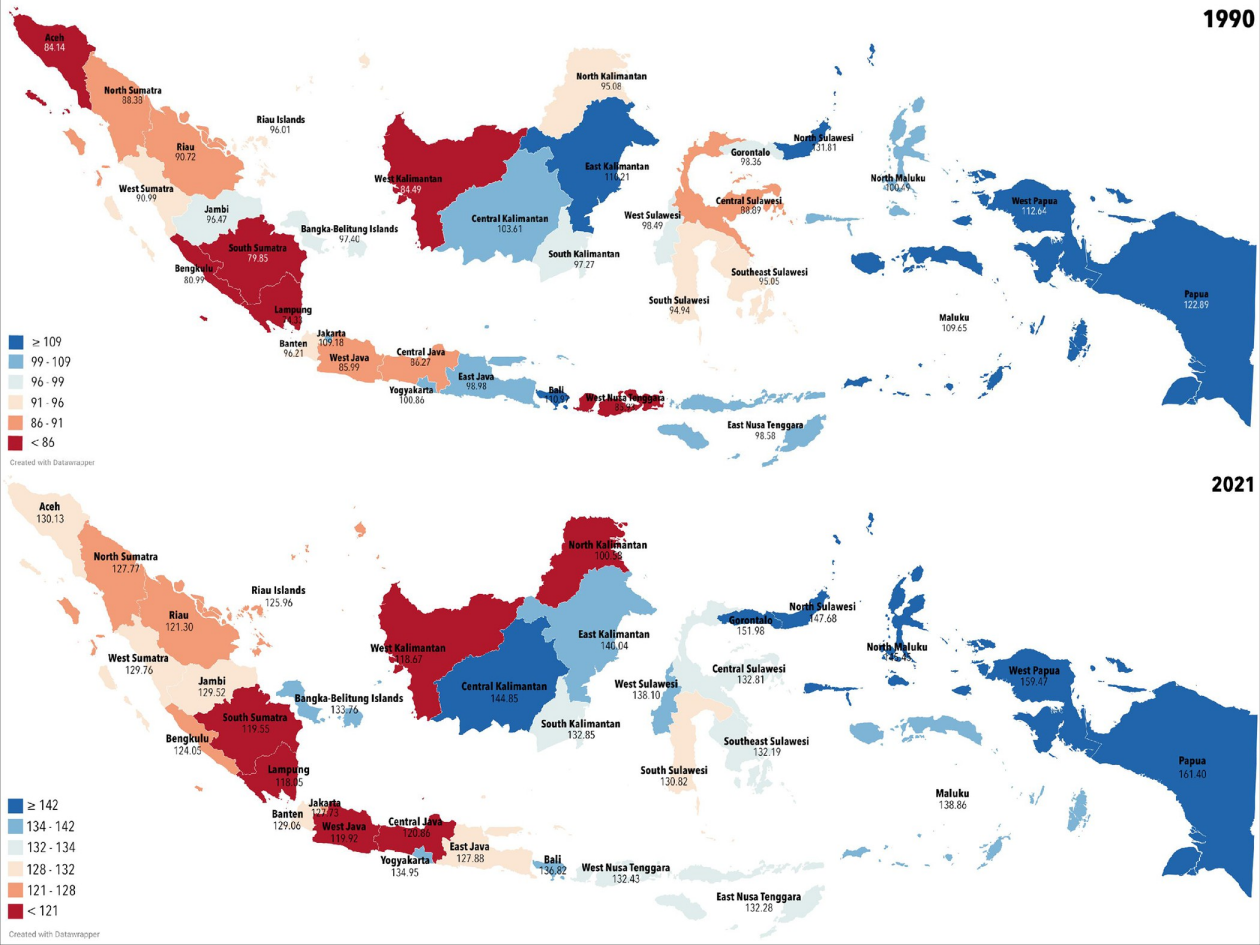
DALYs rate on the provincial level in 1990 and 2021.

The morbidity of OC, as measured by the age-standardized DALYs rate (ASDR) in Indonesia, increased from 92.69 (95% UI, 65.70–171.80) to 126.56 (95% UI, 83.24–210.85), or equal to a change of 36.54% during 1990–2021 (**Table 1, Fig 1**). In 1990, the morbidity of OC was the highest in North Sulawesi at 131.81 (95% UI, 82.33–306.60); however, it shifted to Papua at 161.40 (95% UI, 90.79–382.85) in 2021.

### Burden of OC linked to the leading risk factors

**Fig 2:**
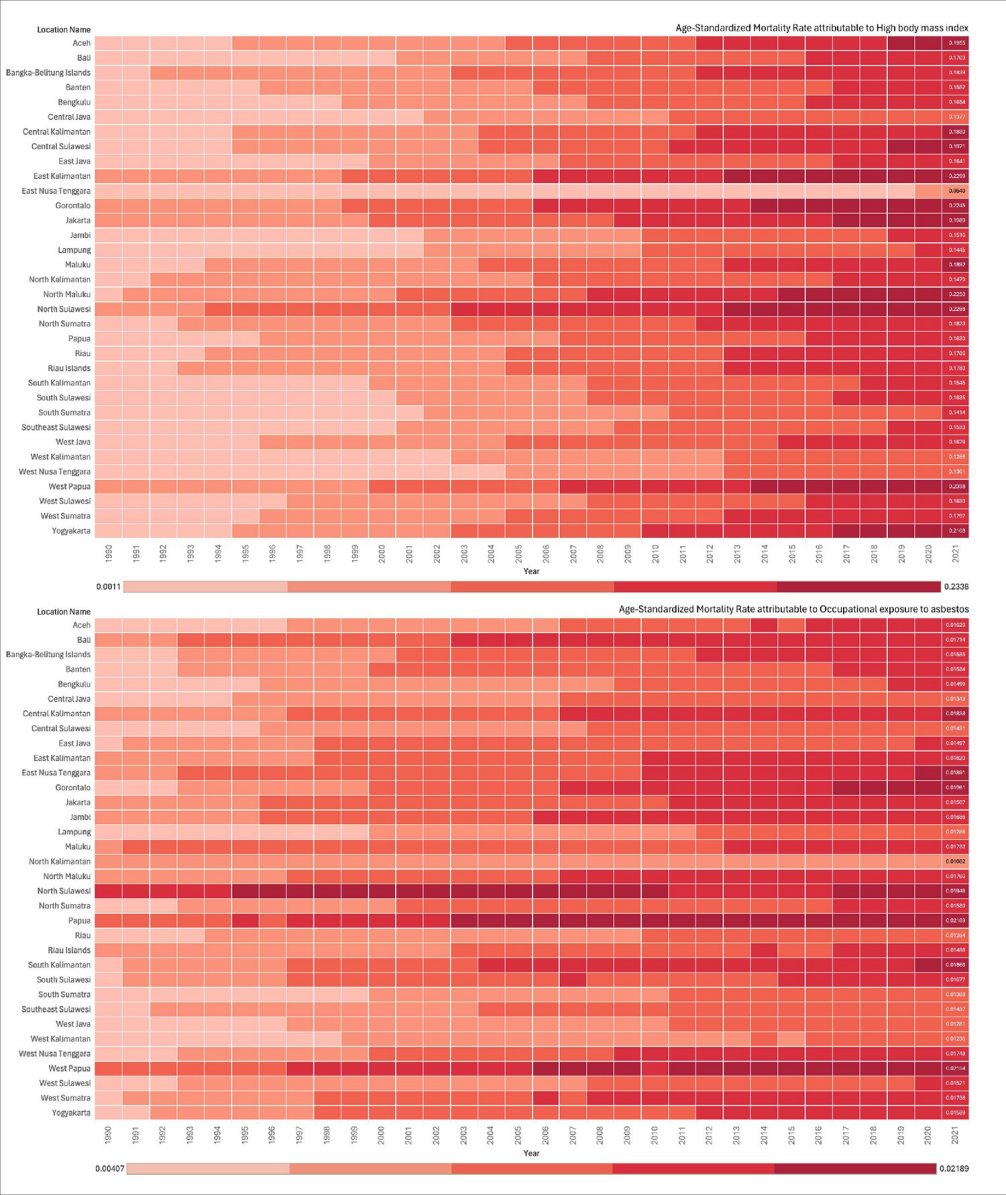
Risk factors associated with ovarian cancer deaths in Indonesia (1990–2021).

In 2021, the ASDR due to all risk factors remained essentially constant from 1990 to 2021, with the ASDR increasing from 1.11 (95% UI, -0.20–2.89) per 100 000 people to 6.19 (95% UI, 1.19–12.93) per 100 000 people. The two risk factors contributing to global deaths due to OC in 1990 were high body mass index and work-related asbestos exposure. These remained the leading risk factors for OC fatalities worldwide in 2021. Additionally, in 2021, among all the risk factors for death due to OC, the risk factor that led to the highest number of deaths was a high body mass index, accounting for 240.23 (95% UI, 35.30–509.91) or an age-standardized death rate of 0.16 (95% UI, 0.02–0.35) per 100 000. Moreover, the ASMR due to all risk factors showed an increase over the last 32 years at 500% from 0.03 (95% UI, -0.01–0.08) to 0.18 (95% UI, 0.04–0.37) (**Fig 2, S3 Fig**).

### SDI in Indonesia

The Indonesian provincial SDIs for 2021 are presented in our article (**S4 Fig).** The average per capita income was $4334 (Indonesian rupiah [IDR] 62.01 million), with a maximum of $19 196 (IDR 274.66 million) in Jakarta and a minimum of $1436 (IDR 20.56 million) in East Nusa Tenggara. In 2021, the average educational attainment of the population older than 15 was 8.97 years. The province with the longest educational attainment was Jakarta (11.2 years), whereas the province with the shortest was Papua (7.05 years). The average number of women of reproductive age (15–49 years) was 73.09 million or 542 per 1000 women. Meanwhile, Indonesia had a total fertility rate of 1.97, slightly lower than Southeast Asia’s rate of 2.05. The 34 provinces were grouped by SDI quartiles: high, high-middle, low-middle, and low, with the highest SDI in Jakarta and the lowest in East Nusa Tenggara (**S4 Fig).**

**Fig 3:**
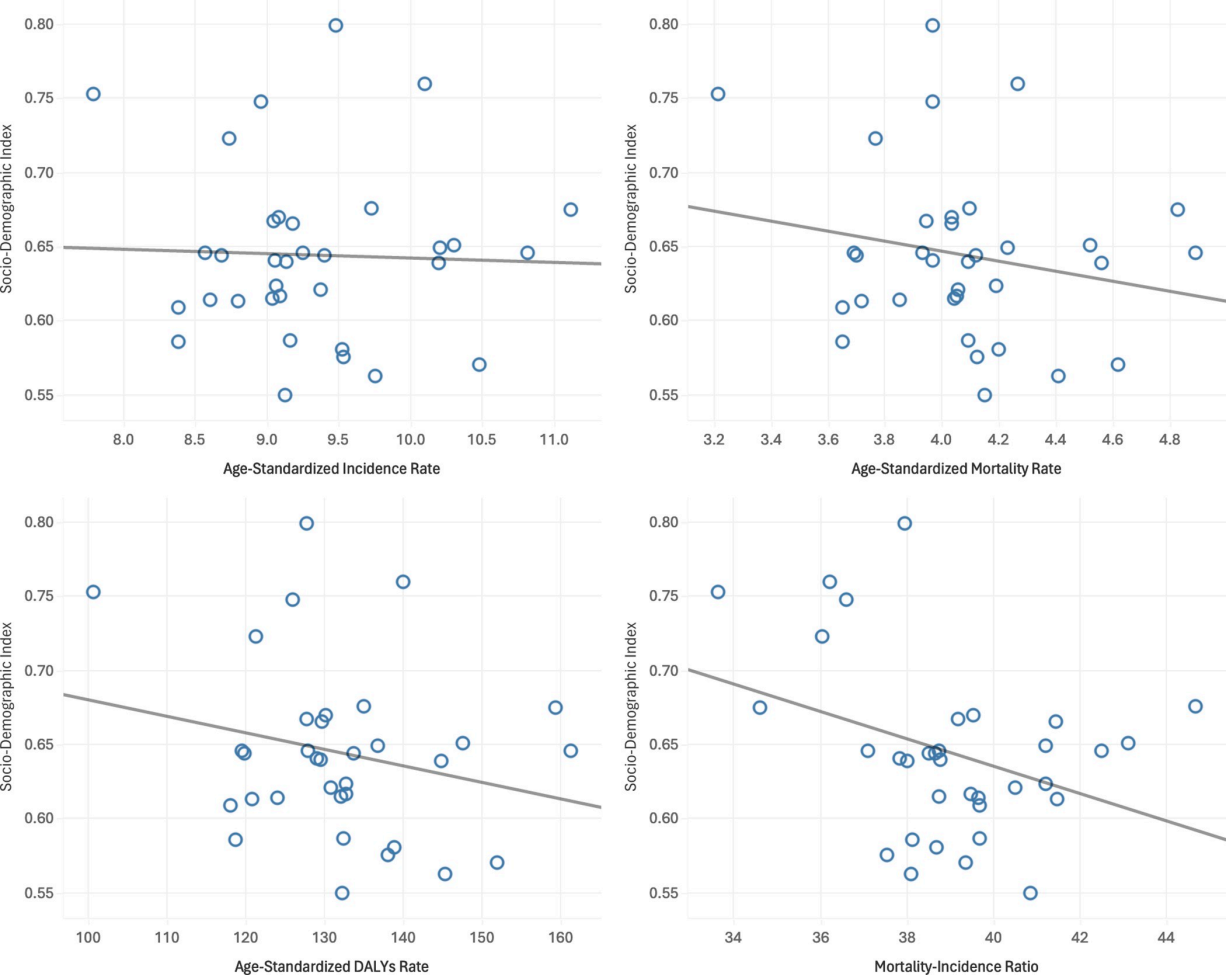
Correlation of SDI and ovarian cancer burden metrics, 2021.

**Fig 3** illustrates the correlation between the SDI and disease burden metrics. There was no statistically significant correlation between SDI and ASIR (Pearson r^2^ = 0.001, p = 0.837; **Fig 3A**), ASMR (Pearson r^2^ = 0.040, p = 0.258; **Fig 3B**) and ASDR (Pearson r^2^ = 0.054, p = 0.187; **Fig 3C**). However, there was a significant negative correlation between SDI and mortality-to-incidence ratio (MIR). (Pearson r^2^ = 0.131, p = 0.035; **Fig 3D**).

**Fig 4:**
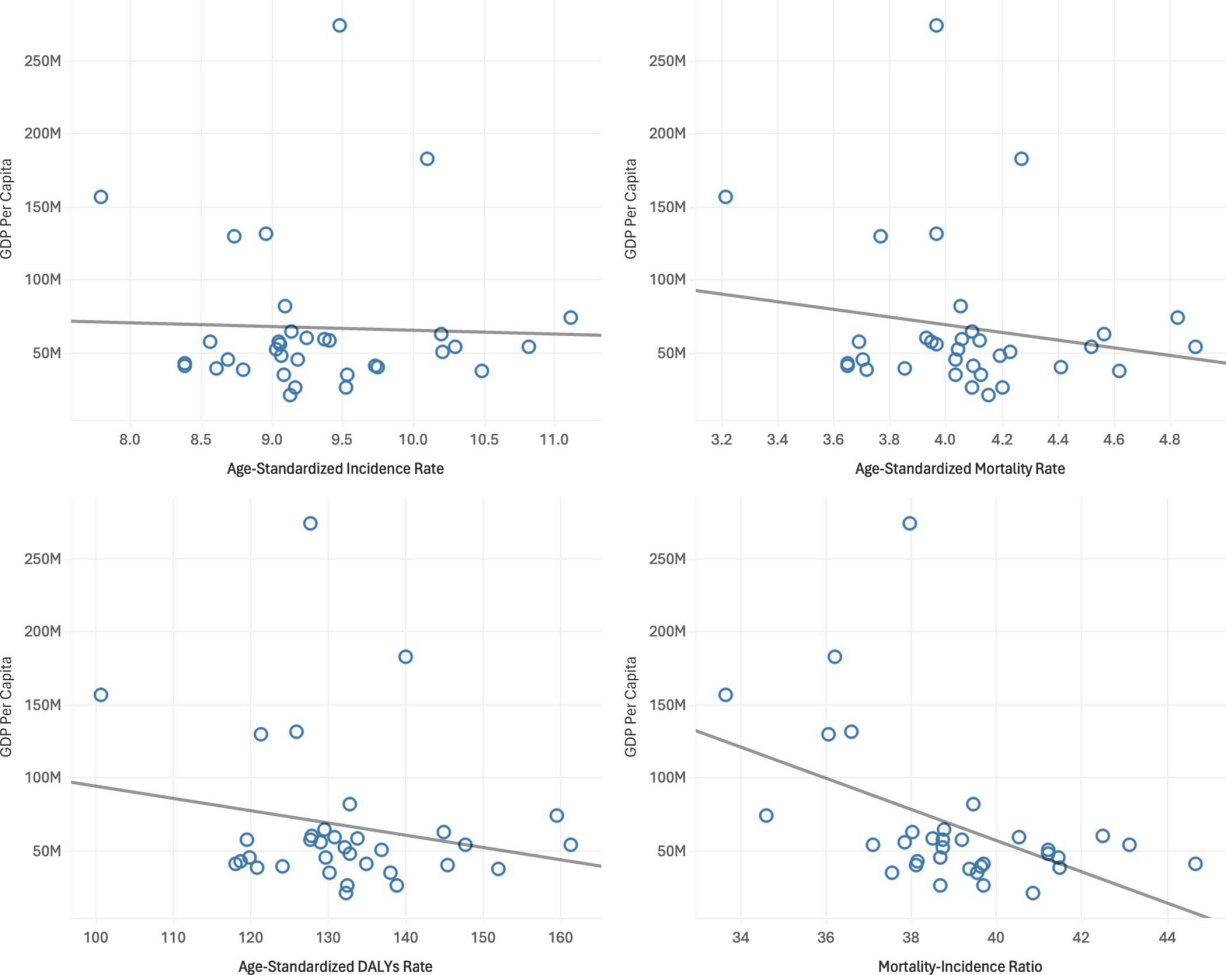
Correlation of GDP per capita and ovarian cancer burden metrics, 2021.

The Indonesian subnational gross domestic product (GDP) per capita at current prices for the 34 provinces statistically correlated with age-standardized OC metrics (**Fig 4**). The higher the GDP, the higher the incidence and burden of disease. There was no statistically significant correlation between GDP and ASIR (Pearson r^2^ = 0.001, p = 0.841; **Fig 4A**), ASMR (Pearson r^2^ = 0.031, p = 0.322; **Fig 4B**) and ASDR (Pearson r^2^ = 0.039, p = 0.264; **Fig 4C**). However, there was a significant negative correlation between GDP per capita and MIR (Pearson r^2^ = 0.223, p = 0.005; **Fig 4D**).

### Coverage of obstetric gynecologists in Indonesia

The number of obstetric gynecologists in Indonesia in 2021 was 4 895 (**S5 Fig**). More than half of the total population (2 734 or 55.9%) resided in Java, especially in the province of Jakarta. The correlation between number of obstetric gynecologists and the number of OC burden metrics at the provincial level is presented in our manuscript (**S6 Fig**). The higher the number of obstetric gynecologists, the higher the number of OC incidence (Pearson r^2^ = 0.778, p < 0.001; **S6A Fig**), number of OC mortality (Pearson r^2^ = 0.760, p < 0.001; **S6B Fig**), and number of OC DALYs (Pearson r^2^ = 0.772, p < 0.001; **S6C Fig**). However, there was no significant correlation between obstetric gynecologists and the MIR of OC (Pearson r^2^ = 0.043, p = 0.241; **S6D Fig**).

### Coverage of oncologic gynecologists in Indonesia

The number of oncologic gynecologists in Indonesia in 2021 was 121 (**S7 Fig**). Nearly half of the total number resided in Java, especially in Jakarta. The number of oncologic gynecologists was positively correlated with the number of OC incidence, mortality, and DALYs at the provincial level in Indonesia (Pearson r^2^ = 0.387, p < 0.001; Pearson r^2^ = 0.382, p < 0.001; and Pearson r^2^ = 0.383, p < 0.001, respectively). However, there was no significant correlation between the number oncologic gynecologists and MIR of OC (Pearson r^2^ = 0.045, p = 0.230) (**S8 Fig**).

## Discussion

This study found 13 250 OC cases and 5 296 deaths in Indonesia in 2021. OC caused 186 917.18 DALYs nationwide, and 179 752.03 from YLLs and 7 165.15 YLDs (**S1 Table**). OC incidence and mortality were higher in Indonesian provinces with middle-high SDIs. Meanwhile, the global incidence and number of deaths of OC in 2019 were 294,422, with 198,412 cases resulting in 5.36 million DALYs [6]. In comparison to our neighbouring country, Indonesia continues to have a greater incidence and mortality of OC, with Malaysia having 1182 new cases, 656 deaths, and 20 093 DALYs and Singapore having 289 new cases, 163 deaths, and 4 478 DALYs in 2019.

Compared to GBD 2017, the incidence of OC has increased from 9 786 to 13 250 (35.4%), associated with risk factors, improved disease registration, and lifestyle changes [23]. Approximately 43.82% of OC new cases occur in middle-high SDI provinces, and a significant proportion of DALYs/YLLs belong to women residing in these provinces. The prevalence of this disease in low-SDI provinces was also notably significant.

According to the GBD, OC mortality and DALYs grew from 1990 to 2021, which has been even greater in recent decades. Although changes in ASIR, ASMR, and ASDR were generally not significant, they showed a significant proportion in areas with a high SDI. This trend is slowly increasing in the low-middle and middle-high SDI regions, consistent with global findings that ASMR increases significantly in the middle, low-middle, and low-SDI regions [23]. Identifying and eliminating health inequalities is crucial for achieving sustainable development, as improved cancer care and treatment directly contribute to reduced mortality rates [24]. Despite this, there are currently no national guidelines in Indonesia for OC screening. Neither the Indonesian Ministry of Health nor the Indonesian Society of Gynecologic Oncologists has established standards for this type of screening. Instead, their focus has been on educating general gynecologists about assessing the malignancy of ovarian tumors and ensuring timely referrals to gynecologic oncology centers.

The Java Island and postmenopausal women have higher rates of OC. This is supported by various Indonesian province studies [25, 26]. Illness risk factors can explain some of these statistical disparities. Moreover, as shown by the risk factors in our study, high body mass index (BMI) was the primary contributor to death due to OC. This is in line with the results of studies showing that BMI, postmenopausal status, age, and parity were significantly higher in diabetic patients with OC than in their counterparts [27]. Moreover, a previous study has established that higher BMI is linked with increased mortality in OC [28]. Higher BMI on the other hand is also associated with increased risk of type 2 diabetes mellitus through the alteration of adipose tissue biology, which in turn connects obesity with insulin resistance and dysfunction of beta cell [29].

Diabetes mellitus negatively impacts the prognosis of OC, reducing both overall survival (OS) and progression-free survival (PFS). Even after adjusting for factors such as age, BMI, cancer stage, histological type, hypertension, menopausal status, and neoadjuvant chemotherapy, patients with diabetes continue to show poorer PFS and OS outcomes [27]. Currently, approximately 240 million people globally have undiagnosed diabetes, and nearly 50% of cases in Southeast Asia go unreported, leaving many unaware of their condition [30]. Controlling diabetes mellitus could be an effective strategy to reduce OC mortality, as well as other complications related to metabolic diseases. Additionally, obesity further worsens the prognosis; studies indicate that obesity worsens survival rates by about 17% in OC patients. Given the rising prevalence of obesity and diabetes, there is an urgent need for community education focused on prevention and early detection.

Asbestos is also widely recognized as a causative factor for OC, although the exact mechanism remains unclear. One hypothesis is that inhaled asbestos fibers are taken up by cells through phagocytosis and subsequently transported via lymphatic channels and mucous membranes, potentially causing inflammation in the ovaries. A meta-analysis by Kim et al. demonstrated a significant association between asbestos exposure and increased mortality from OC [31]. To mitigate the risks associated with asbestos, including OC, it is essential to identify regions with significant asbestos exposure and implement targeted prevention and education programs.

While environmental factors like asbestos increase OC risk, hormonal factors such as oral contraceptive use offer protective benefits. Oral contraceptives reduce the risk across all histological types of OC [24, 25]. Although contraceptive use among married Indonesian women rose modestly from 2007 to 2017, the Indonesia Demographic and Health Survey reported a 12-month discontinuation rate of 29%, with the highest rate observed for pill users [26]. As contraceptive use are associated with number of children, education level, wealth index, and access to information [32], the government should promote contraceptive use, especially oral pills, due to their protective effect against OC [33]. This promotion could be more effective if targeted at specific demographic groups and regions with lower contraceptive use. However, promoting contraceptive use may not be easy, as certain regions in Indonesia hold cultural and religious beliefs that discourage contraception, viewing it as contrary to religious principles [34].

This research serves as a valuable resource highlighting the significance of educating individuals about OC starting at a young age, specifically at the high school level. By doing so, they will be equipped to assume leadership roles within their families, proactively engage in preventive measures, and screen for risk factors associated with OC [35]. Furthermore, this education will encompass other factors that contribute to OC mortality, benefiting both the individuals themselves and their families. The government should implement measures aimed at decreasing the prevalence of OC. While it is not possible to entirely avoid OC, efforts can be made to mitigate its risk factors and those that contribute to mortality. This can be achieved by enhancing public awareness about OC, implementing measures to minimize risk factors, promoting regular screening and and enhancing women’s self-confidence in their ability to identify and respond to symptoms of OC [36].

Compared to studies conducted in other developing countries, particularly in Southeast Asia, this study highlights unique patterns in the incidence, mortality, and overall burden of ovarian cancer (OC) cases within Indonesia. Although direct comparisons are limited by variations in methodologies and data sources, our findings on genetically based OC cases align with broader regional trends. For example, a GLOBOCAN (2020) report noted an increase in OC incidence across several Southeast Asian countries, mirroring the upward trend observed in Indonesia [3]. Similarly, in Singapore, the ASIR of OC rose from 5.8 to 12.5 per 100,000 people per year between 1968 and 2012, even as the ASMR remained stable [37]. The upward trend observed in South and Southeast Asia may influence our findings, potentially reflecting broader demographic shifts due to increased life expectancy and lifestyle changes [38].

### Correlation between the SDI and burden of OC

This study used country-contextualized data sources to measure Indonesia’s SDI at the subnational level in 2021. Provincial SDI metrics were used for 34 Indonesian administrative regions. In 2021, the subnational SDI gap in Indonesia was 0.250 (0.549–0.799), 1.25 times the 1990–2021 difference. Since health determinants affect populations within specific geographic areas, these regions reflect the "space of the risk." Understanding regional health status, therefore, is crucial for identifying and addressing health policy needs at the subnational level [39].

The significant negative correlation between the Sociodemographic Index (SDI) and the Mortality-to-Incidence Ratio (MIR) suggests that higher levels of sociodemographic development are associated with better cancer management outcomes. MIR is a crucial metric for evaluating cancer control and treatment effectiveness across countries, representing the proportion of diagnosed cases that result in death [40]. A lower MIR, indicative of fewer deaths relative to incidence, reflects improved healthcare capabilities, such as timely diagnosis, effective treatments, and access to quality care [41, 42]. Therefore, in regions with higher SDI, better healthcare infrastructure and resources likely facilitate more effective cancer management, resulting in a lower MIR.

Interestingly, the lack of significant correlation between SDI and the Age-Standardized Incidence Rate (ASIR) suggests that sociodemographic factors alone do not heavily influence cancer occurrence. Cancer incidence is shaped by genetic, lifestyle, and environmental factors, which SDI does not fully capture [43]. This implies that even in countries with similar sociodemographic development levels, cancer incidence rates may vary due to these other determinants [44]. Therefore, SDI seems to have a limited role in directly affecting ASIR, emphasizing the complex and multifaceted nature of cancer risk factors [45].

Likewise, the absence of significant correlations between SDI and both ASMR and ASDR indicates that sociodemographic improvements alone may not reduce cancer mortality or the broader disease burden. ASMR and ASDR are influenced by various healthcare-related factors, such as early detection, healthcare access, and palliative care quality, which may not correlate directly with broader sociodemographic indicators like income and education [3]. Therefore, while higher SDI might improve healthcare access, these enhancements may not fully capture the complexities of cancer survival and disease burden [13].

While SDI does not significantly impact cancer incidence or overall mortality, it plays a crucial role in reducing MIR, reflecting better cancer management outcomes in regions with more advanced sociodemographic contexts. Enhancing healthcare systems in lower SDI regions is essential for improving cancer outcomes by reducing MIR. Therefore, targeted efforts to increase healthcare access and quality in these areas are critical for reducing the proportion of deaths relative to cancer incidence, ultimately improving survival rates and supporting the value of MIR as a cancer management effectiveness indicator [40].

### Correlation between the GDP per capita and burden of OC

There are visible disparities in the GDP between provinces. The significant negative correlation between GDP per capita and the MIR suggests that countries with higher economic prosperity can manage cancer more effectively. MIR is widely recognized as an indicator of the effectiveness of cancer care, as it reflects the proportion of diagnosed cases that result in death [40]. Wealthier countries often have better healthcare infrastructure, enabling early diagnosis, advanced treatments, and more accessible healthcare, which collectively contribute to lower MIRs [41, 42].This supports the notion that GDP per capita is crucial in enabling better cancer management outcomes.

The absence of a significant relationship between GDP per capita and ASIR suggests that economic resources do not heavily influence cancer occurrence. Cancer incidence is driven by complex factors, including genetics, lifestyle, and environmental exposures, which are not directly linked to a country’s economic status [43]. As such, cancer incidence appears relatively independent of GDP per capita, emphasizing the multifaceted nature of cancer risk [45].

Similarly, GDP per capita does not directly affect cancer mortality rates or the overall disease burden, as indicated by the lack of correlation with ASMR and ASDR [42]. While economic resources can support healthcare systems, other factors like healthcare efficiency and access play vital roles in outcomes. This highlights that while GDP per capita does not impact cancer incidence, mortality, or burden directly, it is pivotal for reducing MIR, underscoring the importance of economic investment in healthcare infrastructure [13].

### Obstetric gynecologists, oncologic gynecologists, and the burden of OC

This study measured the coverage of obstetric gynecologists (*Sp*. *OG*) and oncologic gynecologists (*Sp*.*OG*., *Subsp*.*Onk*.) and their distributional correlation with the burden of OC at the subnational level in Indonesia in 2021. Despite the high correlation between obstetric gynecologists and important OC outcomes, the MIR did not correlate, which is intriguing. The MIR is an indicator of survival outcomes or prognosis. A higher MIR ratio indicates a lower survival rate. This could imply that provinces with more obstetric gynecologists tend to report more OC cases, possibly because of increased awareness, screening, and detection rather than direct causation.

The greater detection rate may raise reported incidence, mortality, and DALYs, explaining this positive correlation, which aligns with Australia’s findings [46]. However, the lack of a significant correlation between the number of obstetric gynecologists and the MIR ratio is intriguing. A greater MIR indicates poorer patient survival [47]. However, the absence of a correlation with the MIR suggests that additional obstetric gynecologists may increase OC detection and diagnosis but not survival. This has many causes. Late-stage OC diagnosis may explain this. Even though there are more obstetric gynecologists, the prognosis is poor in most OCs diagnosed late due to nonspecific early symptoms or a lack of routine screening [48].

Another consideration is the overall quality of cancer care. The mere presence of more obstetric gynecologists does not guarantee access to high-quality treatment or advanced interventions needed to improve survival rates. This could be due to a lack of resources, limited access to advanced therapeutic options, or deficiencies in training and continuing medical education among healthcare providers. Moreover, geographic disparities in access to healthcare services could also factor into the equation [49]. Although some provinces might have an ample number of gynecologists, others may suffer from severe shortages, leading to a disparity in the quality and accessibility of care that might affect the MIR.

This research serves as a valuable resource highlighting the significance of educating individuals about OC starting at a young age, specifically at the high school level. By doing so, they will be equipped to assume leadership roles within their families, proactively engage in preventive measures, and screen for risk factors associated with OC [48]. Furthermore, this education will encompass other factors that contribute to OC mortality, benefiting both the individuals themselves and their families. The government should implement measures aimed at decreasing the prevalence of OC. While it is not possible to entirely avoid OC, efforts can be made to mitigate its risk factors and those that contribute to mortality. This can be achieved by enhancing public awareness about OC, implementing measures to minimize risk factors like diabetes mellitus and obesity, and promoting regular screening.

In summary, although an increase in the number of obstetric gynecologists may lead to higher detection rates of OC, this does not necessarily equate to improved survival rates. This indicates the need for a more comprehensive approach that boosts the workforce, addresses late-stage diagnoses, enhances the quality of care, and alleviates healthcare disparities. Further research should focus on these areas to develop targeted interventions and policy recommendations.

### Limitations

Our study presents several notable strengths. Firstly, it reveals a consistent rise in both the incidence and mortality of OC since 1990, a trend that is projected to continue across all 34 provinces in Indonesia, with certain geographically distant provinces experiencing a particularly high burden. This underscores the importance of targeted interventions in these high-burden areas. Similar to Indonesia, many Asian nations have witnessed a concerning rise in OC incidence.

Secondly, the study identifies high BMI as the leading specific risk factor for OC deaths in Indonesia, providing a clear target for public health initiatives aimed at reducing OC mortality. Additionally, by correlating socioeconomic and healthcare metrics such as the SDI, GDP per capita, and the number of obstetric and oncologic gynecologists with OC outcomes, the study highlights potential subnational indicators for OC care. These findings can inform policy decisions to improve cancer care and reduce disparities across regions.

However, our study also has some limitations. We relied on data from the GBD study and mathematical models based on surveillance data, rather than primary data sources, which may introduce some biases or inaccuracies. The variability in cancer surveillance systems and systematic reporting between provinces could partly explain the observed differences in OC incidence and mortality rates.

Furthermore, the study did not include clinical staging or detailed histological information, despite the fact that over 90% of OCs arise from the epithelial cells of the ovary, peritoneum, and fallopian tube. Nonetheless, as the first comprehensive study on OC in Indonesia, our research successfully elucidates the rising burden of OC and its attributable risk factors, providing a crucial foundation for future public health strategies and interventions.

## Conclusion

This large-scale epidemiological study described and analyzed the trends and burden of OC at Indonesia’s national and subnational levels. This is the first study on OC at the subnational level and its associated factors in Indonesia. We found that OC incidence, mortality, and DALYs were significantly correlated with the number of healthcare providers, such as obstetric gynecologists and gynecologic oncologists. This finding suggests that OC remains a major global public health concern and is more frequently diagnosed in regions with a higher SDI than a lower SDI. Therefore, targeted and up-to-date preventive strategies for OC are required.

## Supporting information

S1 Fig Incidence number of cases on provincial level in 1990 and 2021. (TIF)

S2 Fig Distribution of ovarian cancer incidences by age group (1990–2021). (TIF)

S3 Fig Risk factors associated with ovarian cancer DALYs in Indonesia (1990–2021). (TIF)

S4 Fig Sociodemographic index at the provincial level in Indonesia in 2021. (TIF)

S5 Fig Distribution of obstetric gynecologists in Indonesia at the provincial level, 2021. (TIF)

S6 Fig Correlation between obstetric gynecologist and disease burden metrics, 2021. (TIF)

S7 Fig Distribution of oncologic gynecologist in provincial level, 2021. (TIF)

S8 Fig Correlation between oncologic gynecologist and disease burden metrics, 2021. (TIF)

S1 Table YLD, YLL, and prevalence of ovarian cancer in Indonesia on provincial level in 1990 and 2021. (DOCX)

S1 Data (CSV)

S2 Data (CSV)

## References

[1] Momenimovahed Z, Tiznobaik A, Taheri S, et al. Ovarian cancer in the world: epidemiology and risk factors. *Int J Womens Health* 2019; 11: 287. doi: 10.2147/IJWH.S197604 31118829 PMC6500433

[2] World Health Organization International Agency for Research on Cancer (IARC). *World Cancer Report*: Cancer Research for Cancer Prevention. IARC, 2020.

[3] Sung H, Ferlay J, Siegel RL, et al. Global Cancer Statistics 2020: GLOBOCAN Estimates of Incidence and Mortality Worldwide for 36 Cancers in 185 Countries. *CA Cancer J Clin* 2021; 71: 209–249. doi: 10.3322/caac.21660 33538338

[4] Rasjad IS, Tjokroprawiro BA. The Role of Procalcitonin as a Prognostic Variable in Ovarian Cancer Patients at Dr. Soetomo General Hospital Surabaya. *Indones J Cancer* 2021; 15: 107–111.

[5] Aziz MF. Gynecological cancer in Indonesia. *J Gynecol Oncol* 2009; 20: 8. doi: 10.3802/jgo.2009.20.1.8 19471661 PMC2676491

[6] Zhang S, Cheng C, Lin Z, et al. The global burden and associated factors of ovarian cancer in 1990–2019: findings from the Global Burden of Disease Study 2019. *BMC Public Health* 2022; 22: 1455. doi: 10.1186/s12889-022-13861-y 35907822 PMC9339194

[7] Reid BM, Permuth JB, Sellers TA. Epidemiology of ovarian cancer: a review. *Cancer Biol Med* 2017; 14: 9–32. doi: 10.20892/j.issn.2095-3941.2016.0084 28443200 PMC5365187

[8] Azizah F, Mulawardhana P, Sandhika W. Association of age at menarche, parity, and hormonal contraceptive use with the histologic type of ovarian cancer. *Maj Obstet Ginekol* 2021; 29: 118–123.

[9] Zhou Z, Wang X, Ren X, et al. Disease Burden and Attributable Risk Factors of Ovarian Cancer From 1990 to 2017: Findings From the Global Burden of Disease Study 2017. *Front public Heal*; 9. Epub ahead of print 17 September 2021. doi: 10.3389/fpubh.2021.619581 34604147 PMC8484795

[10] Vaduganathan M, Mensah GA, Turco JV, et al. The Global Burden of Cardiovascular Diseases and Risk: A Compass for Future Health. *J Am Coll Cardiol* 2022; 80: 2361–2371. 36368511 10.1016/j.jacc.2022.11.005

[11] Yakupu A, Zhang J, Dong W, et al. The epidemiological characteristic and trends of burns globally. *BMC Public Health* 2022; 22: 1–16. 35996116 10.1186/s12889-022-13887-2 PMC9396832

[12] Abbafati C, Abbas KM, Abbasi M, et al. Global burden of 369 diseases and injuries in 204 countries and territories, 1990–2019: a systematic analysis for the Global Burden of Disease Study 2019. *Lancet* 2020; 396: 1204–1222. doi: 10.1016/S0140-6736(20)30925-9 33069326 PMC7567026

[13] Fitzmaurice C, Abate D, Abbasi N, et al. Global, Regional, and National Cancer Incidence, Mortality, Years of Life Lost, Years Lived With Disability, and Disability-Adjusted Life-Years for 29 Cancer Groups, 1990 to 2017: A Systematic Analysis for the Global Burden of Disease Study. 2019; 5: 1749–1768. doi: 10.1001/jamaoncol.2019.2996 31560378 PMC6777271

[14] Stevens GA, Alkema L, Black RE, et al. Guidelines for Accurate and Transparent Health Estimates Reporting: the GATHER statement. *PLOS Med* 2016; 13: e1002056. doi: 10.1371/journal.pmed.1002056 27351744 PMC4924581

[15] Molassiotis A, Kwok SWH, Leung AYM, et al. Associations between sociodemographic factors, health spending, disease burden, and life expectancy of older adults (70 + years old) in 22 countries in the Western Pacific Region, 1995–2019: estimates from the Global Burden of Disease (GBD) Study 2019. *GeroScience* 2022; 44: 925–951. 35000094 10.1007/s11357-021-00494-z PMC9135952

[16] Go DS, Kim YE, Yoon SJ. Subnational Burden of Disease According to the Sociodemographic Index in South Korea. *Int J Environ Res Public Health* 2020; 17: 1–8. 10.3390/ijerph17165788 PMC7460231 32785128

[17] Murray CJL, Callender CSKH, Kulikoff XR, et al. Population and fertility by age and sex for 195 countries and territories, 1950–2017: a systematic analysis for the Global Burden of Disease Study 2017. *Lancet (London*, *England)* 2018; 392: 1995. doi: 10.1016/S0140-6736(18)32278-5 30496106 PMC6227915

[18] Li X, Cao X, Guo M, et al. Trends and risk factors of mortality and disability adjusted life years for chronic respiratory diseases from 1990 to 2017: systematic analysis for the Global Burden of Disease Study 2017. *BMJ*; 368. Epub ahead of print 19 February 2020. doi: 10.1136/bmj.m234 32075787 PMC7190065

[19] Naghavi M, Abajobir AA, Abbafati C, et al. Global, regional, and national age-sex specific mortality for 264 causes of death, 1980–2016: a systematic analysis for the Global Burden of Disease Study 2016. *Lancet (London*, *England)* 2017; 390: 1151–1210. doi: 10.1016/S0140-6736(17)32152-9 28919116 PMC5605883

[20] Yoon SJ, Kim YE, Kim EJ. Why they are different: Based on the burden of disease research of WHO and institute for health metrics and evaluation. *Biomed Res Int*; 2018. Epub ahead of print 23 April 2018. doi: 10.1155/2018/7236194 29850556 PMC5937600

[21] Wyper GMA, Assunção RMA, Colzani E, et al. Burden of Disease Methods: A Guide to Calculate COVID-19 Disability-Adjusted Life Years. *Int J Public Health* 2021; 66: 619011. doi: 10.3389/ijph.2021.619011 34744580 PMC8565264

[22] Datawrapper. Datawrapper, https://www.datawrapper.de/ (2024, accessed 8 June 2024).

[23] Zheng L, Cui C, Shi O, et al. Incidence and mortality of ovarian cancer at the global, regional, and national levels, 1990–2017. *Gynecol Oncol* 2020; 159: 239–247. doi: 10.1016/j.ygyno.2020.07.008 32690392

[24] Patel MI, Lopez AM, Blackstock W, et al. Cancer Disparities and Health Equity: A Policy Statement From the American Society of Clinical Oncology. *J Clin Oncol* 2020; 38: 3439–3448. doi: 10.1200/JCO.20.00642 32783672 PMC7527158

[25] Gea I, Loho M, Wagey F. Gambaran Jenis Kanker Ovarium di RSUP Prof. Dr. R.D. Kandou Manado periode Januari 2013—Desember 2015. *e-Clinic*; 4, 10.35790/ecl.v4i2.14374 (2016).

[26] Mulawardhana P, Hartono P, Nugroho H, et al. Death of 43 Indonesian women with ovarian cancer: A case series. *Int J Surg Case Rep* 2021; 78: 391–396. doi: 10.1016/j.ijscr.2020.12.067 33412408 PMC7803628

[27] Akhavan S, Ghahghaei-Nezamabadi A, Modaresgilani M, et al. Impact of diabetes mellitus on epithelial ovarian cancer survival. *BMC Cancer*; 18. Epub ahead of print 12 December 2018. doi: 10.1186/s12885-018-5162-3 30541490 PMC6291925

[28] Zamorano AS, Hagemann AR, Morrison L, et al. Pre-diagnosis body mass index, physical activity and ovarian cancer mortality. *Gynecol Oncol* 2019; 155: 105–111. doi: 10.1016/j.ygyno.2019.07.025 31383570

[29] Klein S, Gastaldelli A, Yki-Järvinen H, et al. Why does obesity cause diabetes? *Cell Metab* 2022; 34: 11–20. doi: 10.1016/j.cmet.2021.12.012 34986330 PMC8740746

[30] DJ M, EJ B. Global picture—IDF DIABETES ATLAS—NCBI Bookshelf. 10th ed. Brussels: International Diabetes Federation, https://www.ncbi.nlm.nih.gov/books/NBK581940/ (2021, accessed 3 June 2024).

[31] Kim SY, Chang HK, Kwon O, et al. Asbestos Exposure and Ovarian Cancer: A Meta-analysis. *Saf Health Work* 2024; 15: 1–8. doi: 10.1016/j.shaw.2023.11.002 38496274 PMC10944147

[32] Efendi F, Gafar A, Suza DE, et al. Determinants of contraceptive use among married women in Indonesia. *F1000Research 2020 9193* 2020; 9: 193. doi: 10.12688/f1000research.22482.1 32269768 PMC7137393

[33] Avramenko AS, Flanagan JM. An epigenetic hypothesis for ovarian cancer prevention by oral contraceptive pill use. *Clin Epigenetics* 2023; 15: 1–9. 37853473 10.1186/s13148-023-01584-9 PMC10585871

[34] Hasanah E. Java Community Philosophy: More Children, Many Fortunes. *Geneal 2023*, *Vol* 7, *Page* 3 2022; 7: 3.

[35] Suzuki K, Yamanaka M, Minamiguchi Y, et al. Details of Cancer Education Programs for Adolescents and Young Adults and Their Effectiveness: A Scoping Review. *J Adolesc Young Adult Oncol* 2023; 12: 9–33. 35180351 10.1089/jayao.2021.0160

[36] Brain KE, Smits S, Simon AE, et al. Ovarian cancer symptom awareness and anticipated delayed presentation in a population sample. *BMC Cancer* 2014; 14: 171. doi: 10.1186/1471-2407-14-171 24612526 PMC3975332

[37] Hwang JY-FF, Lim W-YY, Tan CS, et al. Ovarian Cancer Incidence in the Multi-Ethnic Asian City-State of Singapore 1968–2012. *Asian Pac J Cancer Prev* 2019; 20: 3563–3569. 10.31557/APJCP.2019.20.12.3563 PMC7173386 31870095

[38] Deo SVS, Sharma J, Kumar S. GLOBOCAN 2020 Report on Global Cancer Burden: Challenges and Opportunities for Surgical Oncologists. *Annals of surgical oncology* 2022; 29: 6497–6500. doi: 10.1245/s10434-022-12151-6 35838905

[39] O’shaughnessy A, Wright J. Healthcare needs assessment. In: Gulliford M, Jessop E (eds) *Healthcare Public Health*. Oxford University Press, pp. 56–68.

[40] Yang TW, Wang CC, Hung WC, et al. Improvement in the Mortality-to-Incidence Ratios for Gastric Cancer in Developed Countries With High Health Expenditures. *Front Public Heal* 2021; 9: 1–8. doi: 10.3389/fpubh.2021.713895 34485236 PMC8415830

[41] Bray F, Ferlay J, Soerjomataram I, et al. Global cancer statistics 2018: GLOBOCAN estimates of incidence and mortality worldwide for 36 cancers in 185 countries. *CA Cancer J Clin* 2018; 68: 394–424. doi: 10.3322/caac.21492 30207593

[42] Allemani C, Matsuda T, Di Carlo V, et al. Global surveillance of trends in cancer survival 2000–14 (CONCORD-3): analysis of individual records for 37 513 025 patients diagnosed with one of 18 cancers from 322 population-based registries in 71 countries. *Lancet (London*, *England)* 2018; 391: 1023–1075. 29395269 10.1016/S0140-6736(17)33326-3 PMC5879496

[43] Ferlay J, Colombet M, Soerjomataram I, et al. Estimating the global cancer incidence and mortality in 2018: GLOBOCAN sources and methods. *Int J cancer* 2019; 144: 1941–1953. doi: 10.1002/ijc.31937 30350310

[44] Wild CP, Weiderpass E, Steward BW. World Cancer Report. World Health Organization, chrome-extension://efaidnbmnnnibpcajpcglclefindmkaj/https://www.iccp-portal.org/system/files/resources/IARC World Cancer Report 2020.pdf (2020).

[45] Siegel RL, Miller KD, Jemal A. Cancer statistics, 2020. *CA Cancer J Clin* 2020; 70: 7–30. doi: 10.3322/caac.21590 31912902

[46] Australian Institute of Health and Welfare. *Cancer in Australia 2021*. Canberra, https://www.aihw.gov.au/getmedia/0ea708eb-dd6e-4499-9080-1cc7b5990e64/aihw-can-144.pdf.aspx?inline=true (2021, accessed 24 November 2023).

[47] Eberth JM, Zahnd WE, Adams SA, et al. Mortality-to-incidence ratios by US Congressional District: Implications for epidemiologic, dissemination and implementation research, and public health policy. *Prev Med (Baltim)* 2019; 129 Suppl: 105849. doi: 10.1016/j.ypmed.2019.105849 31679842 PMC7393609

[48] Lawson-Michod KA, Watt MH, Grieshober L, et al. Pathways to ovarian cancer diagnosis: a qualitative study. *BMC Womens Health* 2022; 22: 1–16. 36333689 10.1186/s12905-022-02016-1 PMC9636716

[49] Graham S, Hallisey E, Wilt G, et al. Sociodemographic disparities in access to ovarian cancer treatment. *Ann Cancer Epidemiol* 2019; 3: 10–10. doi: 10.21037/ace.2019.10.02 32043078 PMC7008774

