# Supplementary figures and images for: Investigation of the trends and associated factors of ovarian cancer in Indonesia: A systematic analysis of the Global Burden of Disease study 1990–2021
Source: PLoS One. 2025 Jan 17;20(1):e0313418. doi: 10.1371/journal.pone.0313418 (PMC11741624; doi:10.1371/journal.pone.0313418)

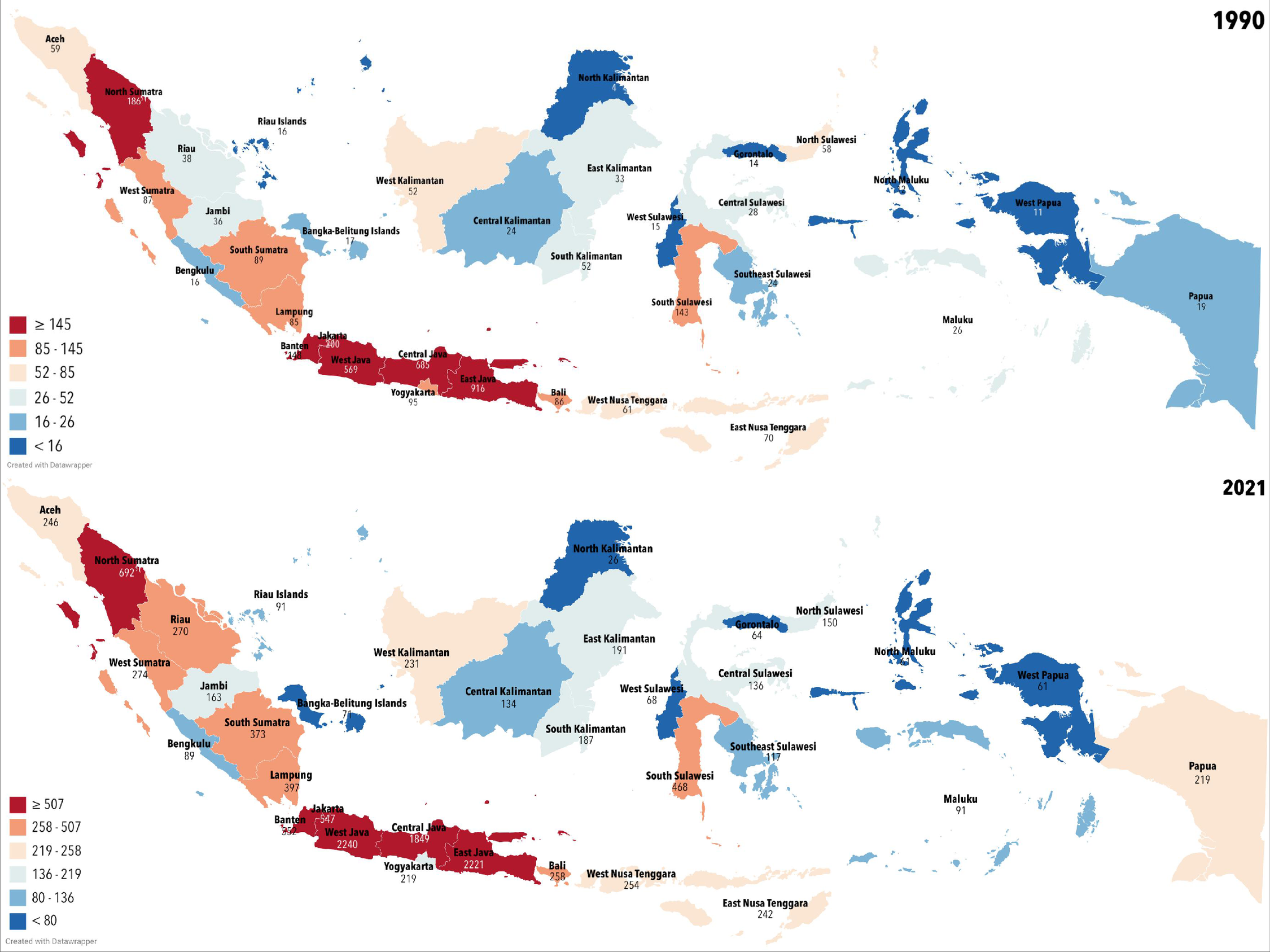

Supplement: S1 Fig — (TIF) [file pone.0313418.s001.tif]

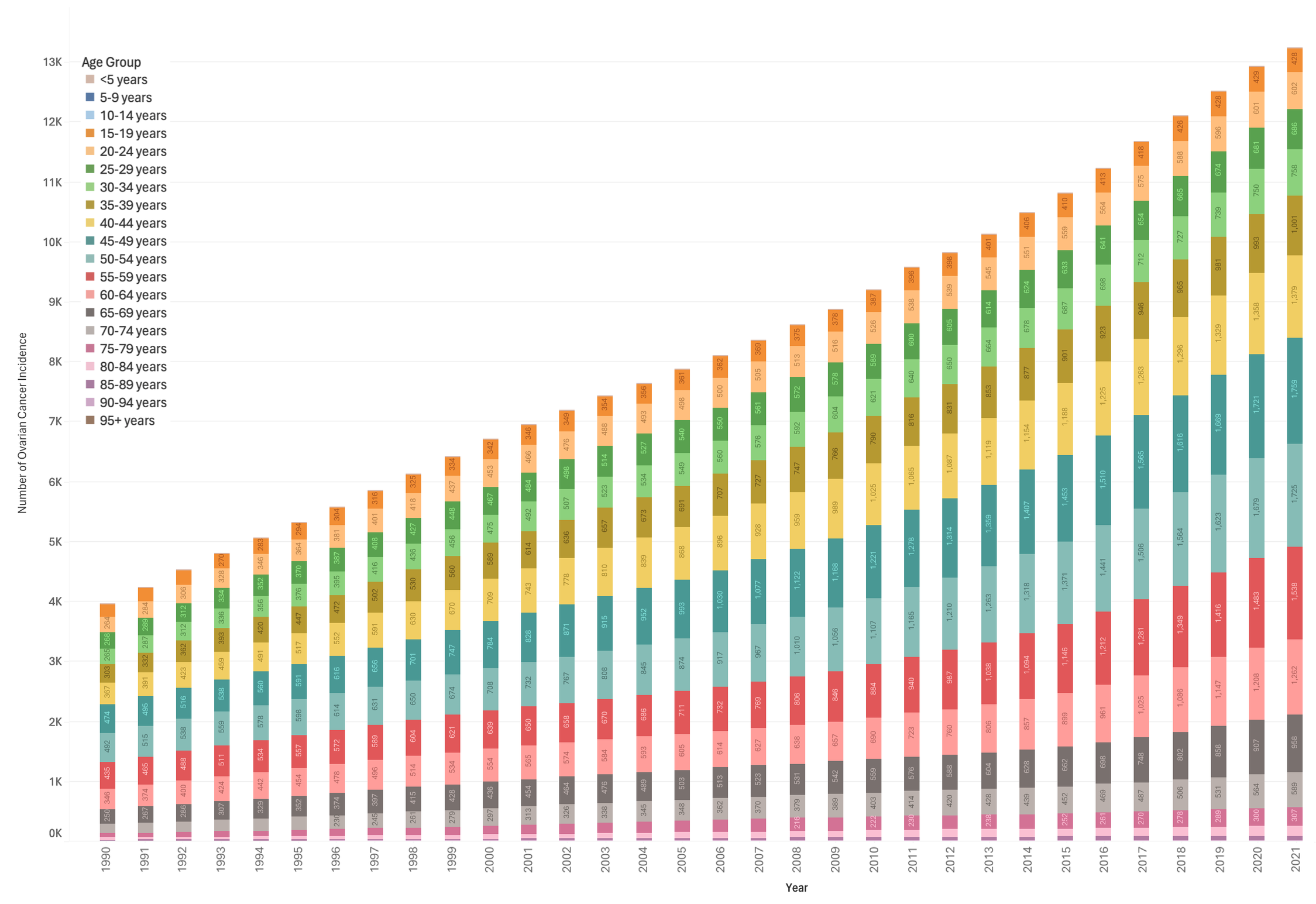

Supplement: S2 Fig — (TIF) [file pone.0313418.s002.tif]

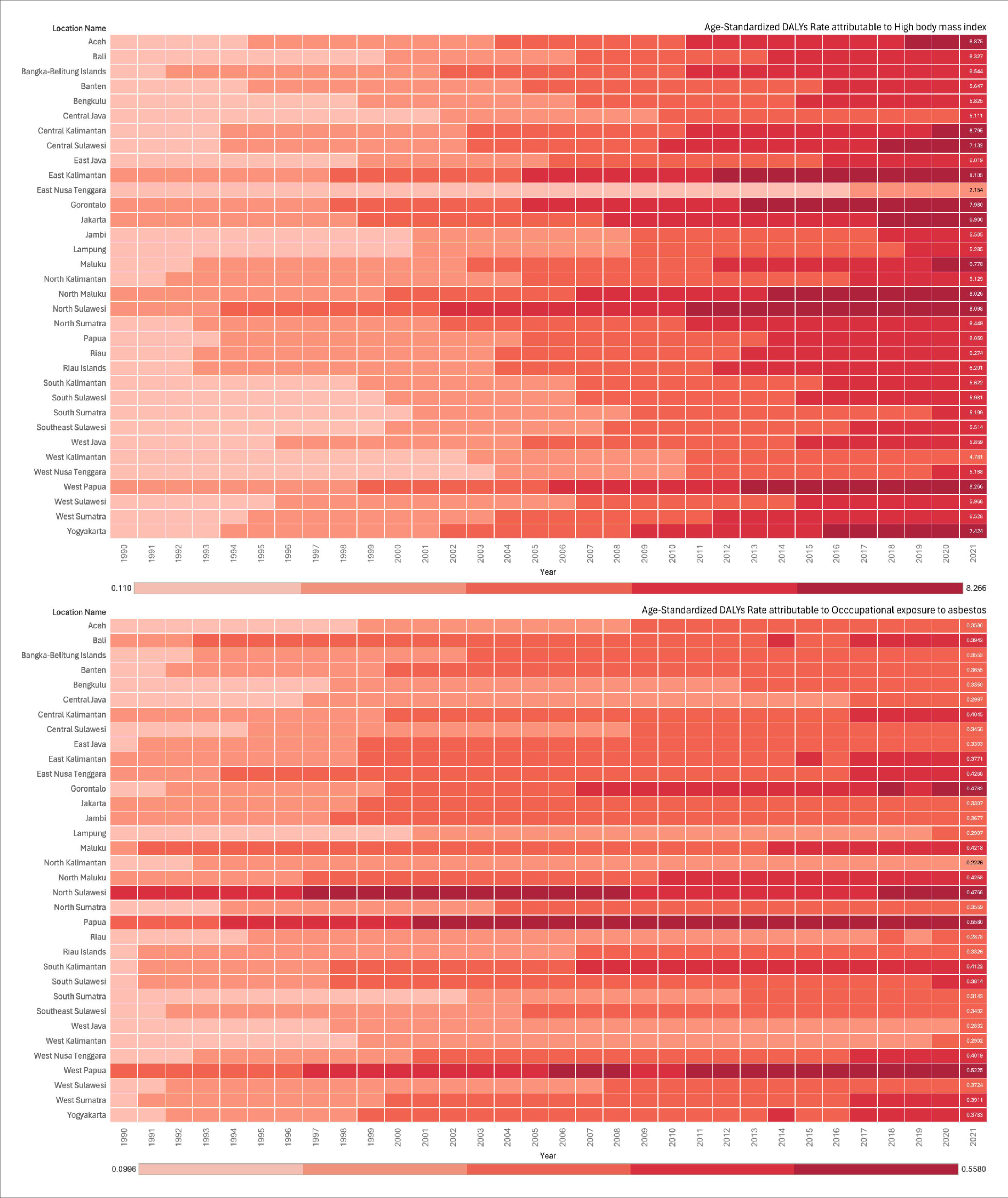

Supplement: S3 Fig — (TIF) [file pone.0313418.s003.tif]

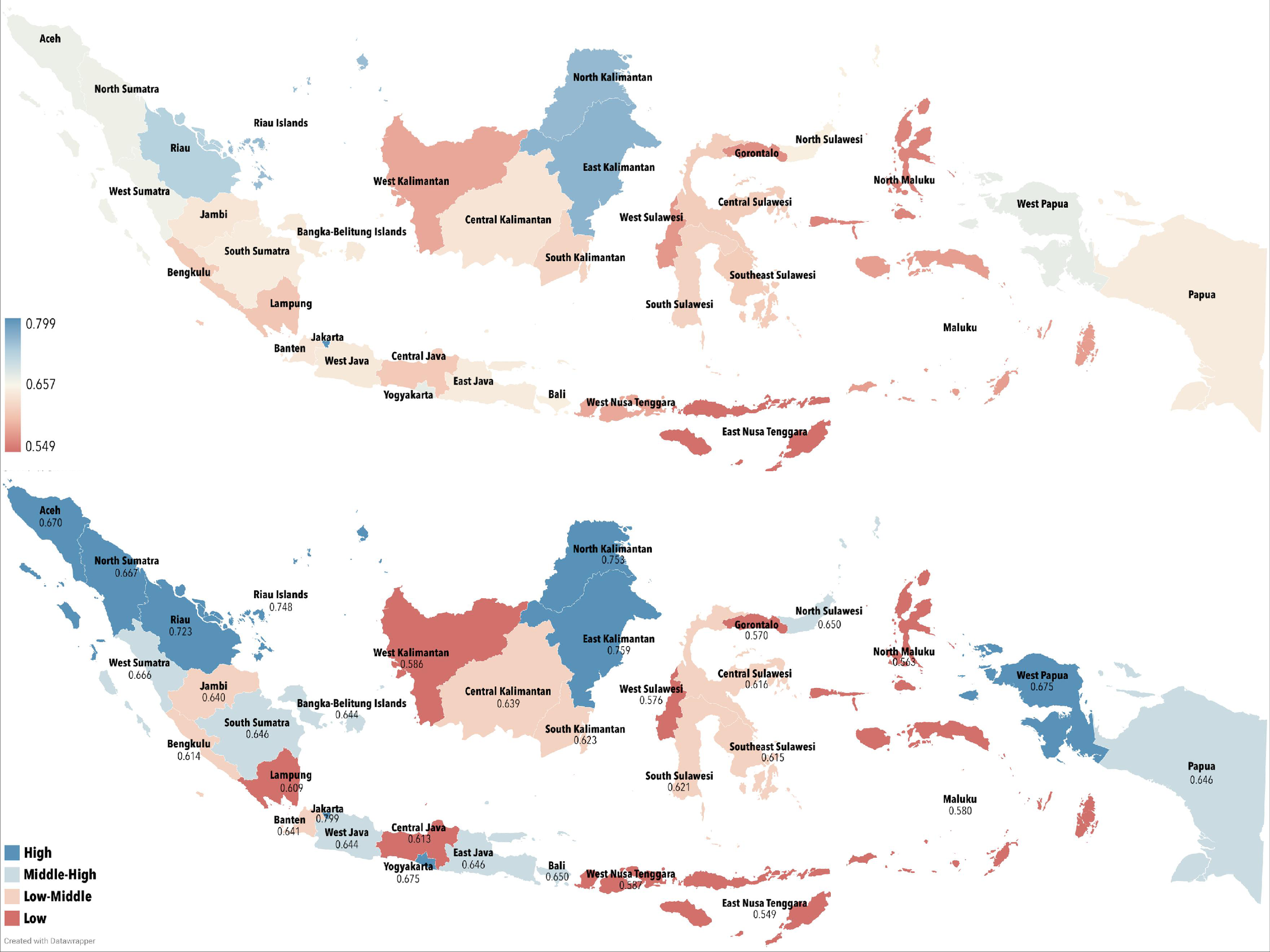

Supplement: S4 Fig — (TIF) [file pone.0313418.s004.tif]

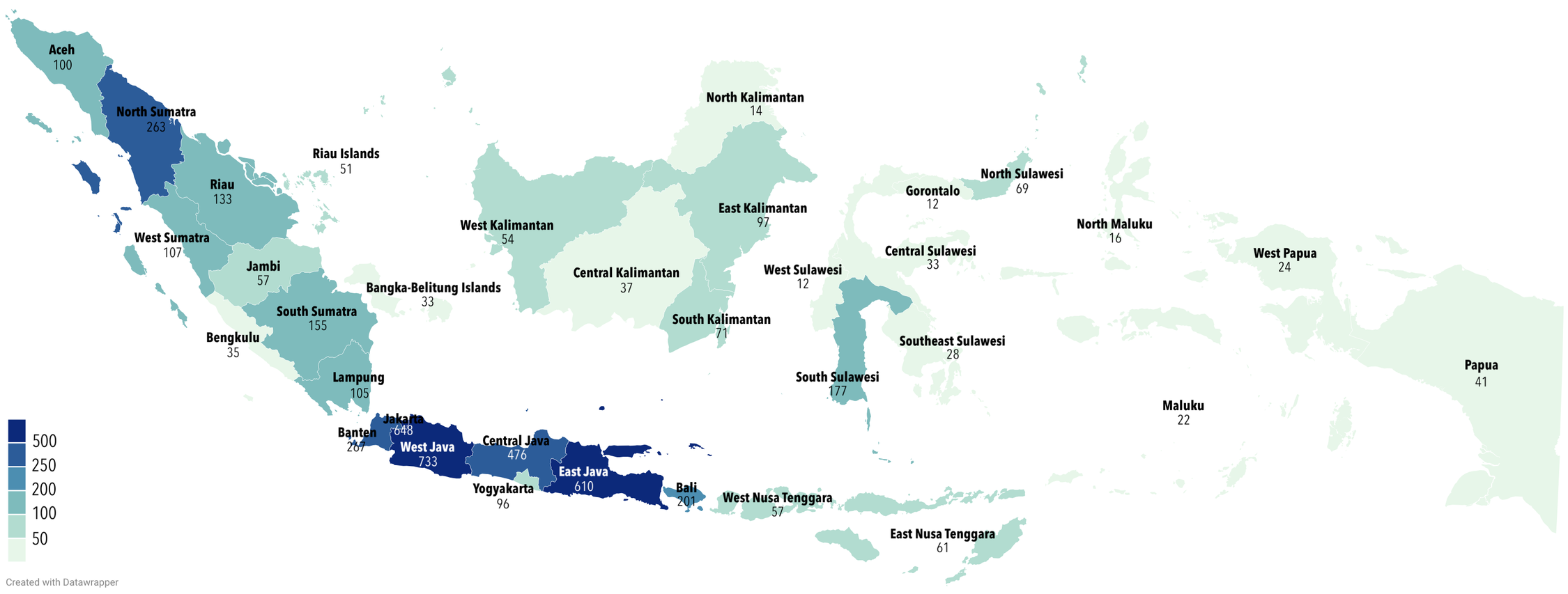

Supplement: S5 Fig — (TIF) [file pone.0313418.s005.tif]

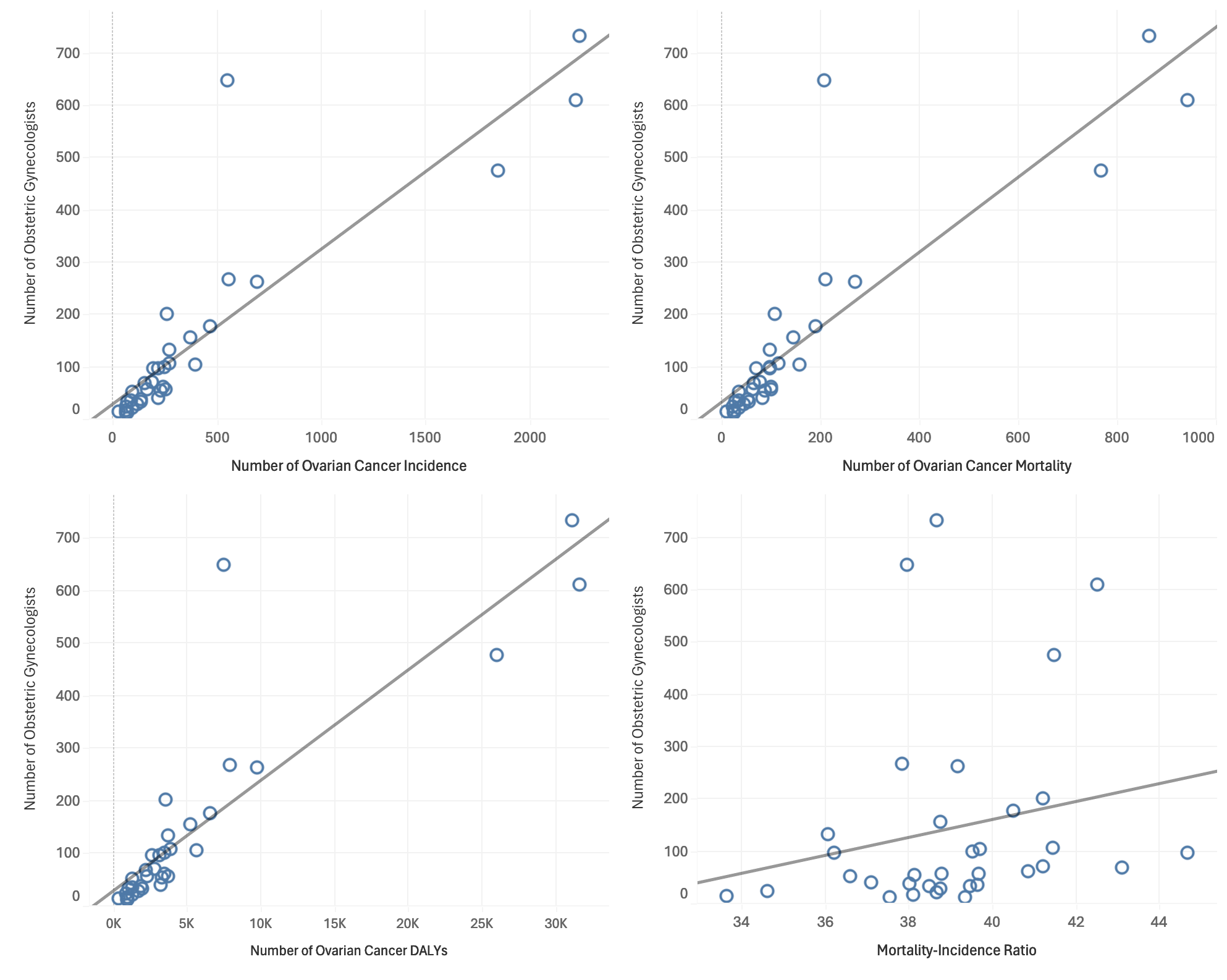

Supplement: S6 Fig — (TIF) [file pone.0313418.s006.tif]

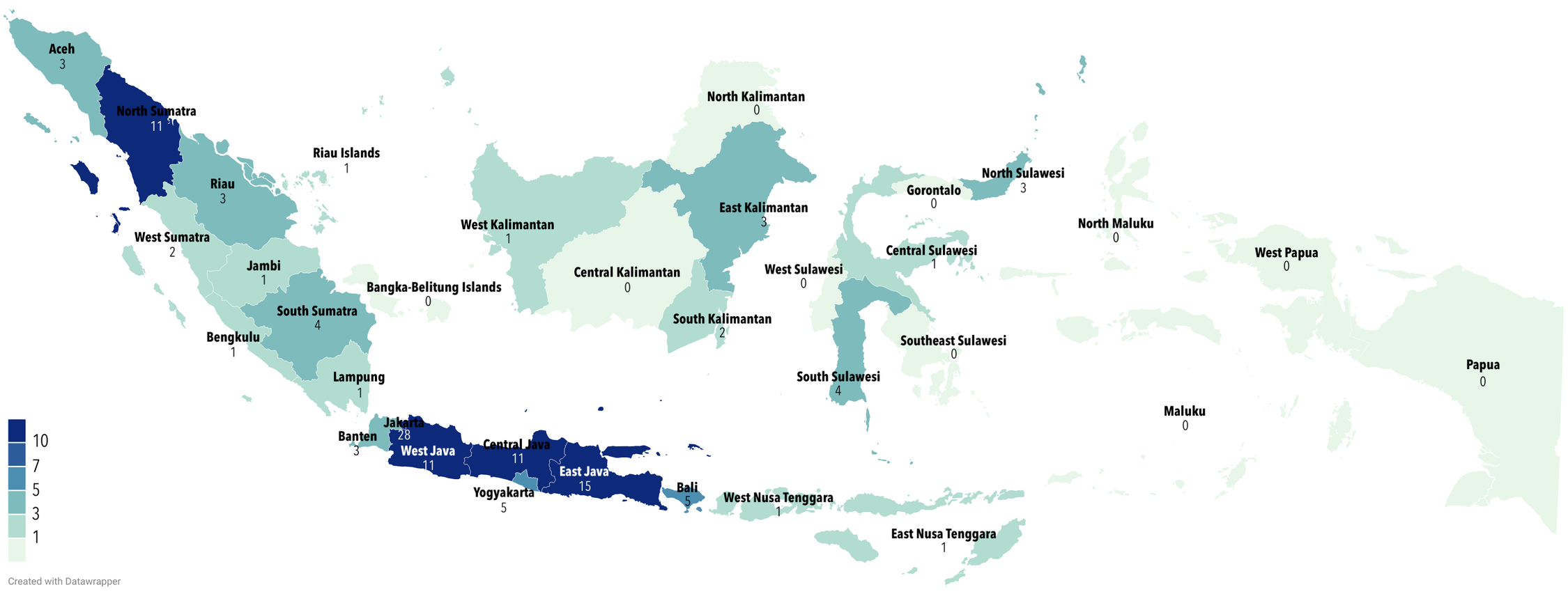

Supplement: S7 Fig — (TIF) [file pone.0313418.s007.tif]

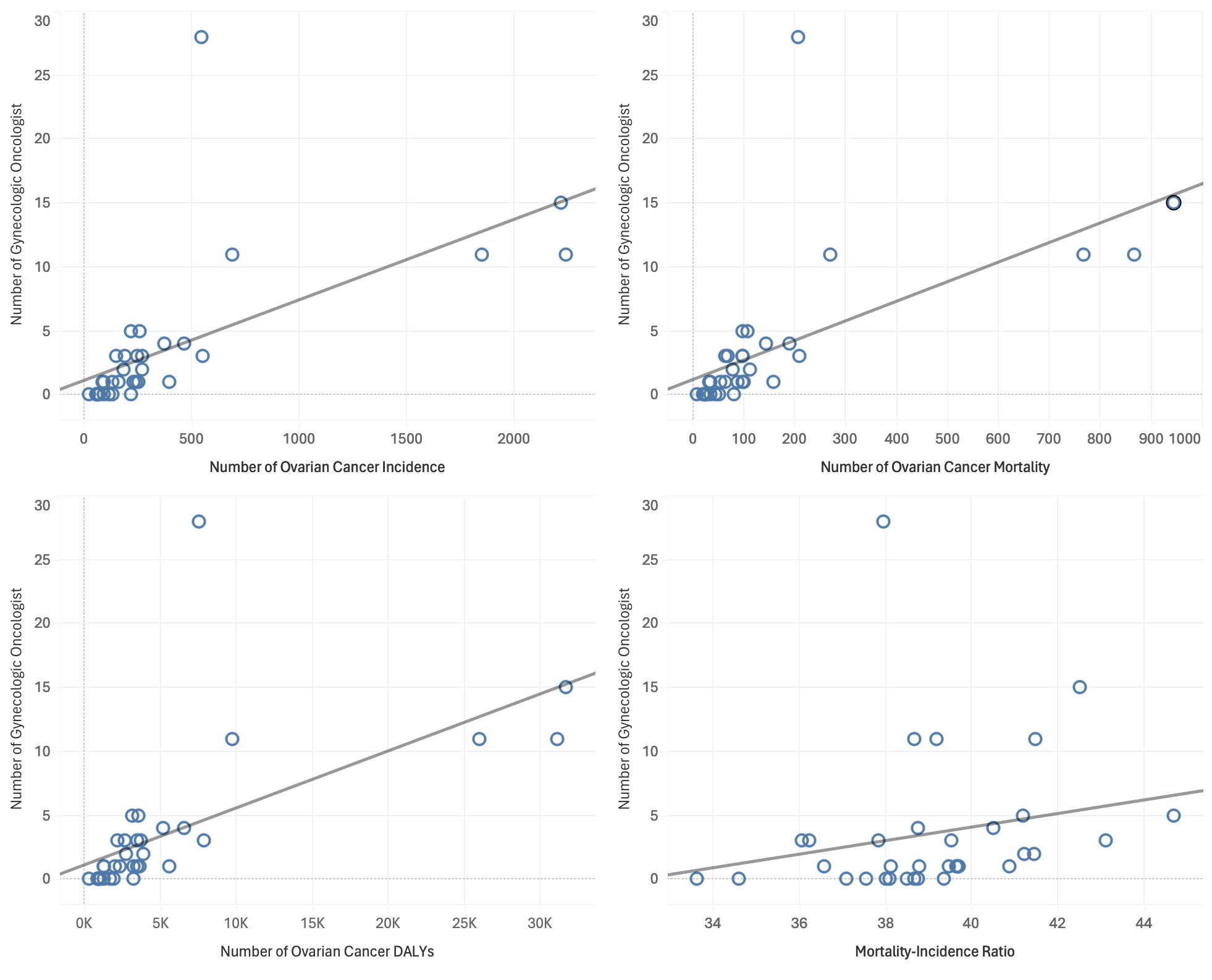

Supplement: S8 Fig — (TIF) [file pone.0313418.s008.tif]
