# Supplementary material for: Investigation of the trends and associated factors of ovarian cancer in Indonesia: A systematic analysis of the Global Burden of Disease study 1990–2021
Source: PLoS One. 2025 Jan 17;20(1):e0313418. doi: 10.1371/journal.pone.0313418 (PMC11741624; doi:10.1371/journal.pone.0313418)
Supplement: S1 Table — (DOCX) [file pone.0313418.s009.docx]

**S1 Table.** YLD, YLL, and Prevalence of Ovarian Cancer in Indonesia on Provincial Level in 1990 and 2021

| **Province** | **Number of YLLs** | | | | **Number of YLDs** | | | | **Number of Prevalence** | | | |
| --- | --- | --- | --- | --- | --- | --- | --- | --- | --- | --- | --- | --- |
|  | **1990** | **2021** | **Change (%)** | **AAPC (%)** | **1990** | **2021** | **Change (%)** | **AAPC (%)** | **1990** | **2021** | **Change (%)** | **AAPC (%)** |
| Indonesia | 58924.48 (41125.70-110428.0) | 179752.03 (117626.12-296832.02) | 205.05 | 3.49 | 2030.48 (1207.01-3860.55) | 7165.15 (4343.36-12436.06) | 252.88 | 3.93 | 17945.42 (12115.59-35035.84) | 63954.69 (652.27-2178.36) | 256.38 | 4.21 |
| Aceh | 873.90 (521.21-1819.95) | 3363.70 (1895.55-6260.55) | 284.91 | 4.27 | 29.91 (15.46-65.57) | 128.83 (67.24-259.94) | 330.73 | 4.54 | 267.60 (154.71-577.25) | 1175.64 (1591.22-4924.10) | 339.33 | 4.87 |
| Bali | 1229.83 (789.69-2698.73) | 3409.85 (1980.31-6036.99) | 177.26 | 3.26 | 44.39 (23.39-99.61) | 142.62 (73.37-261.85) | 221.29 | 3.66 | 392.71 (238.04-879.18) | 1244.96 (729.79-2122.90) | 217.02 | 3.88 |
| Bangka-Belitung Islands | 246.15 (135.83-679.75) | 968.61 (522.70-2224.14) | 293.50 | 4.34 | 8.45 (3.89-24.0) | 37.76 (18.01-92.96) | 346.86 | 4.73 | 74.64 (38.80-212.70) | 340.21 (175.31-815.98) | 355.79 | 4.99 |
| Banten | 2172.74 (1353.25-4128.19) | 7568.93 (4584.94-12702.05) | 248.36 | 3.53 | 75.18 (38.85-153.52) | 301.45 (162.01-558.48) | 300.97 | 3.91 | 701.22 (405.02-1401.78) | 2735.41 (1635.23-4561.12) | 290.09 | 4.47 |
| Bengkulu | 246.97 (135.85-674.13) | 1231.08 (671.79-2641.39) | 398.47 | 5.13 | 8.36 (3.72-22.77) | 47.33 (22.0-100.58) | 466.15 | 5.49 | 72.91 (37.28-206.35) | 421.97 (222.57-871.76) | 478.79 | 5.83 |
| Central Java | 10129.13 (7007.20-16536.71) | 24992.55 (15112.67-43081.0) | 146.74 | 2.84 | 354.0 (199.13-643.92) | 1003.27 (530.11-1829.84) | 183.41 | 3.23 | 3077.70 (1996.03-5112.85) | 8811.55 (5178.01-14475.19) | 186.30 | 3.50 |
| Central Kalimantan | 352.93 (201.03-847.32) | 1819.02 (1076.37-3360.02) | 415.41 | 5.13 | 12.38 (6.09-30.47) | 72.59 (37.12-143.42) | 486.35 | 5.44 | 113.34 (62.01-276.57) | 664.63 (381.75-1209.97) | 486.38 | 5.79 |
| Central Sulawesi | 419.42 (246.54-1089.53) | 1901.25 (1002.41-4303.55) | 353.30 | 4.83 | 14.01 (6.57-37.94) | 71.0 (34.08-166.72) | 406.78 | 5.14 | 124.34 (67.77-334.92) | 638.19 (332.89-1487.90) | 413.26 | 5.44 |
| East Java | 14026.95 (9375.17-27116.46) | 30456.09 (17274.84-51082.93) | 117.13 | 2.39 | 463.58 (246.89-898.54) | 1202.56 (645.05-2080.34) | 159.41 | 2.95 | 3950.60 (2531.23-7661.40) | 10399.29 (5963.20-17523.32) | 163.23 | 3.25 |
| East Kalimantan | 454.01 (247.79-1281.80) | 2527.0 (1243.63-6050.17) | 456.60 | 5.62 | 17.17 (7.61-51.98) | 103.84 (47.73-235.99) | 504.78 | 5.81 | 164.57 (84.51-490.23) | 963.46 (464.47-2444.43) | 485.43 | 5.90 |
| East Nusa Tenggara | 1104.01 (643.34-2299.96) | 3330.43 (1977.18-5344.11) | 201.67 | 3.44 | 35.50 (17.06-76.96) | 127.11 (67.72-219.60) | 258.06 | 3.94 | 294.25 (161.73-645.71) | 1132.83 (638.41-1889.15) | 284.99 | 4.40 |
| Gorontalo | 214.28 (122.71-566.66) | 893.41 (470.81-2138.56) | 316.94 | 4.79 | 7.21 (3.46-19.55) | 33.73 (15.40-83.47) | 367.82 | 5.14 | 62.61 (33.64-182.71) | 300.13 (150.45-766.96) | 379.34 | 5.24 |
| Jakarta | 2638.54 (1621.66-5227.33) | 7196.48 (3983.16-13656.70) | 172.74 | 3.20 | 103.55 (52.37-220.26) | 303.98 (162.07-564.44) | 193.56 | 3.33 | 1036.28 (593.54-2201.33) | 2761.03 (41198.19-101490.11) | 166.44 | 3.28 |
| Jambi | 514.01 (290.65-1257.26) | 2220.26 (1266.85-4395.28) | 331.95 | 4.63 | 18.35 (8.80-46.60) | 87.30 (44.27-162.98) | 375.75 | 4.88 | 170.04 (90.10-437.54) | 790.93 (442.61-1531.68) | 365.13 | 5.07 |
| Lampung | 1246.99 (792.62-1889.05) | 5394.23 (3216.17-8314.11) | 332.58 | 4.59 | 44.25 (24.97-74.11) | 213.54 (117.81-353.25) | 382.58 | 4.94 | 397.83 (239.28-622.76) | 1921.66 (1131.27-3011.92) | 383.03 | 5.21 |
| Maluku | 392.14 (205.64-1020.82) | 1254.31 (704.60-2681.53) | 219.86 | 3.42 | 12.94 (5.58-35.57) | 47.68 (22.99-96.87) | 268.47 | 3.77 | 113.58 (52.36-310.40) | 434.30 (228.18-920.93) | 282.39 | 4.30 |
| North Kalimantan | 55.62 (34.17-84.49) | 310.75 (145.39-520.91) | 458.70 | 5.81 | 2.32 (1.24-3.78) | 14.89 (6.02-26.21) | 541.81 | 6.22 | 23.06 (13.70-36.03) | 143.48 (63.45-242.26) | 522.33 | 6.17 |
| North Maluku | 187.08 (95.74-537.19) | 865.67 (449.42-2276.11) | 362.73 | 4.85 | 6.10 (2.43-18.09) | 32.05 (14.40-85.45) | 425.41 | 5.22 | 52.78 (23.85-155.78) | 290.68 (146.87-792.42) | 450.71 | 5.54 |
| North Sulawesi | 859.05 (522.40-2029.99) | 2116.03 (1259.21-4034.59) | 146.32 | 2.68 | 29.21 (15.51-70.49) | 78.62 (40.87-142.71) | 169.15 | 2.86 | 258.69 (146.21-627.89) | 684.29 (395.39-1312.05) | 164.52 | 3.21 |
| North Sumatra | 2740.08 (1761.26-5292.02) | 9365.41 (5666.34-16669.42) | 241.79 | 3.84 | 94.23 (48.84-182.30) | 374.79 (200.02-656.65) | 297.74 | 4.28 | 846.26 (508.01-1719.60) | 3380.93 (1981.34-6030.41) | 299.52 | 4.55 |
| Papua | 266.04 (124.70-733.93) | 3127.85 (1735.09-7544.05) | 1075.71 | 8.25 | 9.47 (3.66-27.43) | 115.81 (58.0-282.01) | 1122.91 | 8.28 | 90.31 (35.59-255.36) | 1057.89 (567.54-2726.73) | 1071.40 | 8.04 |
| Riau | 551.92 (334.31-1264.75) | 3552.90 (2029.82-6490.08) | 543.73 | 6.22 | 19.79 (10.12-47.77) | 149.82 (77.83-283.0) | 657.05 | 6.72 | 179.65 (101.23-416.74) | 1384.02 (774.18-2430.47) | 670.39 | 6.81 |
| Riau Islands | 229.81 (135.21-575.01) | 1222.0 (689.77-2592.07) | 431.74 | 5.12 | 8.25 (4.05-22.28) | 50.89 (26.01-106.54) | 516.85 | 5.61 | 76.17 (41.96-193.80) | 462.57 (253.18-928.34) | 507.27 | 5.95 |
| South Kalimantan | 794.54 (474.52-1911.48) | 2672.70 (1569.63-5488.30) | 236.38 | 3.82 | 26.32 (13.09-67.41) | 97.64 (46.82-213.81) | 270.97 | 4.10 | 230.19 (130.30-589.14) | 861.83 (483.16-1831.10) | 274.40 | 4.42 |
| South Sulawesi | 2146.31 (1337.33-4440.80) | 6291.64 (3849.08-10940.0) | 193.14 | 3.35 | 72.40 (40.08-154.10) | 250.73 (136.30-445.91) | 246.31 | 3.88 | 630.55 (367.44-1420.22) | 2235.82 (1363.09-3921.86) | 254.58 | 4.19 |
| South Sumatra | 1269.21 (828.14-2156.05) | 5004.71 (2991.73-7968.07) | 294.32 | 4.42 | 45.80 (26.03-85.50) | 201.92 (103.16-359.14) | 340.87 | 4.75 | 421.05 (258.40-741.39) | 1833.13 (1058.96-3021.18) | 335.37 | 4.87 |
| Southeast Sulawesi | 364.62 (203.06-1022.88) | 1619.09 (888.71-3420.97) | 344.05 | 4.71 | 12.10 (5.45-33.87) | 60.94 (28.44-132.18) | 403.64 | 5.09 | 107.11 (54.85-316.61) | 556.10 (290.85-1235.74) | 419.19 | 5.43 |
| West Java | 8337.54 (5591.66-15427.20) | 29913.67 (18912.29-48452.27) | 258.78 | 4.07 | 292.30 (166.81-560.53) | 1222.91 (657.86-2097.64) | 318.37 | 4.52 | 2612.51 (1689.41-5088.78) | 11079.64 (6689.02-18499.18) | 324.10 | 4.80 |
| West Kalimantan | 776.15 (431.33-1893.83) | 3119.76 (1810.58-6047.90) | 301.95 | 4.36 | 27.24 (13.81-68.88) | 125.89 (65.34-248.17) | 362.15 | 4.79 | 242.34 (129.57-602.61) | 1140.57 (637.06-2196.35) | 370.65 | 5.05 |
| West Nusa Tenggara | 943.03 (520.28-2415.11) | 3539.62 (2020.34-7060.32) | 275.35 | 4.17 | 31.07 (14.30-80.12) | 135.09 (68.70-264.64) | 334.79 | 4.63 | 267.01 (137.89-718.41) | 1208.27 (682.39-2393.80) | 352.52 | 4.99 |
| West Papua | 150.23 (65.01-444.05) | 810.07 (401.06-2187.72) | 439.22 | 5.25 | 5.59 (1.95-16.95) | 32.66 (13.66-86.48) | 484.26 | 5.42 | 53.99 (19.47-154.08) | 306.50 (141.07-816.01) | 467.65 | 5.46 |
| West Sulawesi | 224.57 (115.76-685.90) | 923.17 (493.91-2017.32) | 311.08 | 4.56 | 7.28 (3.16-23.87) | 35.90 (17.60-87.68) | 393.13 | 5.05 | 63.57 (31.06-198.81) | 327.66 (170.42-746.70) | 415.46 | 5.38 |
| West Sumatra | 1346.43 (877.46-2620.53) | 3735.51 (2194.43-6164.97) | 177.44 | 3.21 | 43.52 (22.97-83.94) | 144.57 (76.81-255.81) | 232.19 | 3.79 | 366.0 (224.73-767.36) | 1281.90 (747.38-2101.41) | 250.25 | 4.15 |
| Yogyakarta | 1420.27 (889.63-3139.29) | 3034.28 (1794.17-5647.45) | 113.64 | 2.40 | 48.28 (24.74-105.67) | 115.43 (59.40-216.18) | 139.08 | 2.67 | 409.94 (246.66-901.54) | 983.22 (554.56-1773.46) | 139.84 | 3.02 |

In the table, dark red signifies metrics substantially above, and dark blue significantly below, the national average; lighter shades of red and blue indicate slight deviations. Grey indicates metrics at or near the national average.
